# Connectomic insight into unique stroke patient recovery after rTMS treatment

**DOI:** 10.3389/fneur.2023.1063408

**Published:** 2023-07-06

**Authors:** Rong Chen, Nicholas B. Dadario, Brennan Cook, Lichun Sun, Xiaolong Wang, Yujie Li, Xiaorong Hu, Xia Zhang, Michael E. Sughrue

**Affiliations:** ^1^The First Affiliated Hospital of Hainan Medical University, Haikou, Hainan, China; ^2^Robert Wood Johnson Medical School, Rutgers University, New Brunswick, NJ, United States; ^3^Xijia Medical Technology Company Limited, Shenzhen, China; ^4^International Joint Research Center on Precision Brain Medicine, XD Group Hospital, Xi'an, Shaanxi, China; ^5^Omniscient Neurotechnology, Sydney, NSW, Australia; ^6^Cingulum Health, Sydney, NSW, Australia

**Keywords:** rTMS, connectomic, DTI, fMRI, networks, stroke, motor

## Abstract

An improved understanding of the neuroplastic potential of the brain has allowed advancements in neuromodulatory treatments for acute stroke patients. However, there remains a poor understanding of individual differences in treatment-induced recovery. Individualized information on connectivity disturbances may help predict differences in treatment response and recovery phenotypes. We studied the medical data of 22 ischemic stroke patients who received MRI scans and started repetitive transcranial magnetic stimulation (rTMS) treatment on the same day. The functional and motor outcomes were assessed at admission day, 1 day after treatment, 30 days after treatment, and 90 days after treatment using four validated standardized stroke outcome scales. Each patient underwent detailed baseline connectivity analyses to identify structural and functional connectivity disturbances. An unsupervised machine learning (ML) agglomerative hierarchical clustering method was utilized to group patients according to outcomes at four-time points to identify individual phenotypes in recovery trajectory. Differences in connectivity features were examined between individual clusters. Patients were a median age of 64, 50% female, and had a median hospital length of stay of 9.5 days. A significant improvement between all time points was demonstrated post treatment in three of four validated stroke scales utilized. ML-based analyses identified distinct clusters representing unique patient trajectories for each scale. Quantitative differences were found to exist in structural and functional connectivity analyses of the motor network and subcortical structures between individual clusters which could explain these unique trajectories on the Barthel Index (BI) scale but not on other stroke scales. This study demonstrates for the first time the feasibility of using individualized connectivity analyses in differentiating unique phenotypes in rTMS treatment responses and recovery. This personalized connectomic approach may be utilized in the future to better understand patient recovery trajectories with neuromodulatory treatment.

## 1. Introduction

Stroke has remained a leading cause of death worldwide which has increased in both incidence and prevalence over recent decades (1, 2). Of the patients who survive, few make a complete recovery and most patients are left with significant disability (3). Despite this, many patients remain highly open to rigorous recovery treatments and training services to improve the quality of life and integration back into society (4, 5), and as such, neurological rehabilitation treatments to facilitate functional recovery after stroke have remained a key priority in stroke research (1). In particular, an improved understanding of the neuroplastic potential of the human brain connectome has facilitated increased use of non-invasive neuromodulatory treatments for stroke patients (1, 6–11).

Non-invasive neuromodulatory treatment, delivered through transcranial magnetic stimulation (TMS), is a recognized and safe treatment that works primarily through modulating cortical and corticospinal excitability across the human cerebrum. While a number of studies in the literature have suggested clear benefits of this therapy in regard to post-stroke functional recovery (7, 12, 13), these benefits have also been contested in recent large scale studies suggesting limited improvements (1, 14). Notably, differences in outcomes across controlled trials may be related to differences in the recovery scale utilized (15, 16), the specific neuromodulatory protocols and targets selected (17, 18), and importantly, unique inter-individual differences in patient physiology (19). Nonetheless, a poor understanding of the variable responses to TMS treatments has disbarred the effective application and recommendation of this safe treatment for stroke patients in larger clinical and research settings (1), and thus requires further study.

It has become clear that human physiological and pathophysiological functioning can be best understood in the context of underlying neural connections across the human brain connectome (8, 20, 21). More recently, these connections can now be rigorously analyzed with the recent advancements in neuroimaging capabilities and high-throughput approaches (22). Similar to what has been seen in a number of other neurological disorders (20, 23), connectomic analyses have revealed that stroke disrupts structural and functional neural connections both near and spatially distant from the lesion site (24, 25), and these disruptions are highly related to functional outcomes (19, 26). This has caused some to suggest the need for a connectomic-based approach to stroke treatments and analyses (27).

It is also important to consider that stroke patient recovery varies significantly between individuals (19). A connectome-based TMS approach that considers individual connectivity disturbances post-craniotomy can facilitate effective improvements in motor and speech deficits for individual brain tumor patients (11). Therefore, it is reasonable to hypothesize that similar patient-specific connectomic analyses may offer additional novel information to understand and predict individual recovery from stroke (19). Utilization of this information may help track the patient recovery course following acute stroke, which could assist in physician decisions regarding treatment parameters and regimens by stratifying patients into different TMS treatment recovery groups (9, 11).

In this pilot study, we attempted to examine how patients could be grouped into specific clusters according to their clinical treatment phenotypes, and how connectomic information may provide additional important insight into understanding these phenotypes.

## 2. Methods

### 2.1. Participants

The study was completed with the first affiliated hospital of Hainan medical university ethics committee approval. Twenty-two patients with acute strokes provided informed consent to the use of rTMS treatment from 2020 to 2021.

Inclusion criteria included: ① being between the ages of 18 and 90; ② having the first and unilateral onset within 1 week; ③ being able to cooperate with physical examination, scoring, and treatment; ④ met the diagnostic criteria of the 2018 China guidelines for the diagnosis and treatment of acute ischemic stroke, as confirmed by cranial CT or MRI; and ⑤ were diagnosed with infarct lesions in the cerebral hemisphere. Exclusion criteria included: ① hemorrhage stroke and progressive stroke; ② intravenous thrombolysis or vascular interventional therapy; ③ metal or foreign matter in the body; and ④ other important organ failure, intracranial hypertension symptoms, or malignant tumor.

### 2.2. Functional outcome assessment

Appropriate demographic data and relevant medical history were collected from each patient. Patient functional status scores were assessed according to: (1) National Institutes of Health Stroke Scale (NIHSS), which is an 11-item neurological examination stroke scale used to evaluate the effect of acute cerebral infarction on the levels of consciousness, language, neglect, visual-field loss, extraocular movement, facial palsy, motor strength, ataxia, dysarthria, and sensory loss. The total scores range from 0 to 42, with higher scores indicating greater severity. (2) Fugl-Meyer Assessment (FMA) is a 5-domain and 155-item scale to assess motor functioning, balance, sensation, and joint functioning in patients with post-stroke hemiplegia at all ages. Each item is scored by a 3-point ordinal scale, with lower scores indicating greater severity. (3) Barthel Index (BI), which is a 10-item scale describing the activities of daily living (ADL) and mobility, and includes 10 personal activities: feeding, personal toileting, bathing, dressing and undressing, getting on and off a toilet, controlling bladder, controlling bowel, moving from wheelchair to bed and returning, walking on a level surface (or propelling a wheelchair if unable to walk), and ascending and descending stairs. Total scores are 100, with lower scores indicating greater dependency. (4) Wolf Motor Function Test (WMFT) includes 15 task performances to measure the upper extremity function after stroke. The total score is 75 with a higher score indicating stronger ability to complete the upper limb tasks (28–31). Each patient's scores were assessed at four-time points in order to obtain long-term data: (1) at admission day, (2) 1 day after treatment, (3) 30 days after treatment, and (4) 90 days after treatment. All the personally identifiable information has been removed. There were no adverse and unanticipated events reported.

### 2.3. Image acquisition

Imaging acquisition was performed within after 48–72 h after the functional outcome assessment and was performed on a Philips 3T Achieva MRI scanner. Diffusion-weighted imaging (DWI) was acquired with: 2 × 2 × 2 mm^3^ voxels, field of view (FOV) = 256 mm, matrix = 128 × 128 mm^2^, slice thickness = 2.0 mm, one non-zero *b*-value of 1,000, 40 directions, gap = 0.0 mm. Resting-state functional MRI (rs-fMRI) was acquired as a T2-star EPI sequence, with 3 × 3 × 3-mm^3^ voxels, 128 volumes/run, TE = 27 ms, TR = 2.8 s, FOV = 256 mm, flip angle = 90°. The sequence time is 230 s. The patient was requested to close their eyes without thinking or any movement during the scan.

### 2.4. rTMS treatment

rTMS treatment was performed the day after imaging acquisition. rTMS was delivered daily, and the patients were treated twice a day for 5 days, a total of 10 times throughout the hospital stay.

The rTMS was performed with a TMS stimulator (YINGCHI Technology, China) using a flat circular coil for accurately targeted stimulation. The coil were placed tangentially to the scalp with the handle posterior at 45° from the mid-line. In order to record surface electromyography (EMG), electrodes were placed on the abductor pollicis brevis (AFB) on the unaffected side. Resting motor threshold (RMT) is defined as the minimum intensity required eliciting at least five out of 10 MEPs that are >50 μV in a relaxed target muscle. The coil positioning was guided throughout a positioning cap with pre-defined brain regions.

Patients were randomly divided into three intervention groups using an automated random lot drawing technique. Based on randomization, patients received different TMS treatments as described in Table 2. The three treatment options were selected based on previous rTMS evidence-based guidelines that recommended that low-frequency or high-frequency TMS could be used as a Class A or B recommendation for the treatment of post-stroke motor dysfunction in the acute (subacute) stage (32). While less stated in previous guidelines, intermittent theta burst stimulation (iTBS) has also been shown to provide benefits in this context with sustained benefits for at least 3 months and therefore was also utilized in our study (33, 34). Information on the TMS protocol used in the current study is presented in Table 1.

**Table 1:** TMS protocols.

**TMS protocol**	**Motor threshold**	**Stimulation frequency (Hz)**	**Trains**	**Pulses/ train**	**Intervals between trains (s)**	**Total pulses**	**Duration (Min)**	**Side**	**Target**	***N* = 22**
iTBS	80%	5 Hz burst frequency, 3 pulses/burst at 50 Hz pulses frequency	20	30	8	600	3	Ipsilesional	M1	5 (23%)
High-frequency	90%	10 Hz	100	10	10	1,000	18	Ipsilesional	M1	10 (45%)
Low-frequency	90%	1 Hz	100	10	2	1,000	20	Contralesional	M1	7 (32%)

### 2.5. MRI image processing

All MRI scans were processed using Infinitome software (produced by Omniscient Neurotechnology), which has been described previously (23, 35). Diffusion tractography preprocessing includes standard processing steps (36), which include motion correction, elimination of excess movement, gradient distortion correction, eddy correction, and constrained spherical deconvolution-based deterministic tractography. An individualized, parcellated brain connectome was then created according to the Human Connectome Project (37) parcellation scheme, and structural connectivity is measured between each parcel pair. Resting-state fMRI image preprocessing steps include similar steps as outlined above in addition to the removal of high variance confounds according to the CompCor method and the regression of motion confounds out of the image and spatial smoothing (38).

### 2.6. Statistical analyses

Analyses were completed using R 4.1.3 (R Foundation for statistical computing).

Data were analyzed for mean or median for continuous variables and as frequency or percentages for categorical data. Continuous variables were assessed for normality with the Shapiro–Wilk's test and homogeneity of variance with the *F*-test of variance and then subsequently compared with unpaired *t*-tests or Wilcoxon rank-sum tests (with Bonferroni correction for multiple comparisons) and univariate linear regression analysis as appropriate. Categorical variables were assessed with chi-squared tests with Yate's continuity correction or Fisher's exact tests as appropriate. Paired subjects at different time points [(1) at admission day, (2) 1 day after treatment, (3) 30 days after treatment, and (4) 90 days after treatment] were assessed using the non-parametric Friedman's test for all four scales. The effect size for possible differences was measured with Kendall's *W* and Dunn's pairwise *post hoc* analyses.

### 2.7. Structural and functional connectivity analyses

After completing tractography-based individual patient connectomes, structural and functional connections between parcels in the motor network were assessed.

Possible structural connectivity disturbances in the cortical-spinal tracts (CSTs), cortical–subcortical projection fibers, and subcortical connections were assessed according to their structural integrity on a 3-point scale (0 = intact, 1 = visible injured, and 2 = absent) as well as the lesion proximity to these structures (0 = not adjacent, 1 = adjacent (<1 cm), and 2 = inside the fibers). These structural connectivity analyses were completed by two independent reviewers (YZ and MES) similar to what has been completed by others (39).

Functional connectivity disturbances within the motor network were assessed by identifying individual “anomaly” parcels, referring to regions functioning outside of the normal range compared to 200 healthy adults. The source of the data is from healthy subjects of similar but not age-matched adults from the publicly available OpenNeuro (https://openneuro.org/) and SchizConnect (http://schizconnect.org) datasets as previously discussed by our team (35, 40). The personalized atlas created in previous steps was registered to the T1 image and localized to the gray matter regions. Although the entire human connectome according to the atlas published by the Human Connectome Project authors demonstrates a total of 360 cortical parcellations (37) as well as an additional 19 subcortical structures (35), we sought to focus on the motor network and subcortical regions alone. Therefore, in the current study, the average BOLD time series from parcellations confined to the motor network and subcortical structures were extracted, including a total of 45 regions (see the details of 45 regions in Supplementary Table 1). In order to create individual functional connectivity anomaly matrices that identify outliers (“anomalies”), a tangent space connectivity matrix was performed to determine the range of each functional connectivity pair in the matrix and create an individual raw functional connectivity matrix. Then, anomaly matrices were created by identifying abnormally connected parcels defined as a 3-sigma outlier for that correlation compared to the normative connectivity matrix. Connections that were 3-SD above the normative mean were labeled “hyperconnected,” within 3-SD labeled “normal connectivity,” and 3-SD below the mean “hypoconnected” (23). Furthermore, the highest variance 1/3 of pairs were excluded to further reduce the false discovery rate. This was based on the hypothesis that since these areas had the highest inter-subject variance in a normal cohort, these areas may be more prone to false discovery and therefore should be excluded, as previously elucidated elsewhere (23, 41).

### 2.8. Hierarchical clustering

An unsupervised machine learning algorithm was utilized to group patients into similar, unique clusters according to their recovery profile and treatment response. Namely, an agglomerative hierarchical clustering method was utilized which groups objects into clusters based on their similar characteristics in a “bottom up” approach (42, 43). Each node (object) represents a cluster, and then clusters are subsequently merged based on their dis(similarity) until the optimal number of clusters K is obtained. Information about (dis)similarity between clusters is calculated using the pairwise Euclidean distances between every pair of clusters in a data matrix. The optimal number of clusters K based on this distance information is then determined according to the Silhouette method. In brief, a Silhouette coefficient, which presents a metric to calculate the goodness of a clustering technique, is obtained and ranges between −1 and 1, with higher scores representing more coherent clusters. Mathematically, it models the difference between cluster separation and cohesion in order to identify the optimal quality of clustering according to a specific number of clusters generated (44).

The individual features utilized in the algorithm included the individual stroke scale scores at four-time points (pre-TMS at baseline and 1-day, 30-day, and 90-day post-TMS). These values were chosen for the current clustering analysis in order to identify individual phenotypes in recovery trajectory (45), rather than identifying clinical presentation phenotypes first and then subsequently assessing their relevance to treatment responses (46). Importantly, we completed this clustering technique for each individual scale separately. This was done secondary to the observation that combining elements from each scale into the same analysis on this relatively small cohort with heterogenous data resulted in poor statistical fitting consisting of clustering into more than 14 groups of 1–2 patients per cluster.

## 3. Results

The 22 patients included in the study were of a median (IQR) age of 64 (56, 68) years, and split equally of male (*n* = 11) and female (*n* = 11) patients. All patients suffered from a stroke, and the median (IQR) hospitalization duration was 9.5 (9, 11) days. The stroke most occurred in the right hemisphere (*n* = 15, 68%). The average baseline score on the NIHSS scale was 11.1, on FMA 16.5, on BI 8.9, and on WFMT 11.8. These data are presented in Table 2.

**Table 2:** Demographics by stroke scale and cluster.

**Characteristic**	**All data**	**NIHSS cluster**							**FMA clusters**			**BI cluster**						**WMFT clusters**		
	***N*** = **22**^a^	**1**, ***N*** = **4**^a^	**2**, ***N*** = **3**^a^	**3**, ***N*** = **8**^a^	**4**, ***N*** = **4**^a^	**5**, ***N*** = **2**^a^	**6**, ***N*** = **1**^a^	***p*** **-value** ^b^	**1**, ***N*** = **18**^a^	**2**, ***N*** = **4**^a^	***p*** **-value** ^c^	**1**, ***N*** = **2**^a^	**2**, ***N*** = **5**^a^	**3**, ***N*** = **6**^a^	**4**, ***N*** = **3**^a^	**5**, ***N*** = **6**^a^	***p*** **-value** ^b^	**1**, ***N*** = **16**^a^	**2**, ***N*** = **6**^a^	***p*** **-value** ^c^
**Lesion side**																				
Left	7 (32%)	1 (25%)	1 (33%)	3 (38%)	2 (50%)	0 (0%)	0 (0%)	>0.9	6 (33%)	1 (25%)	>0.9	0 (0%)	3 (60%)	1 (17%)	2 (67%)	1 (17%)	0.3	5 (31%)	2 (33%)	>0.9
Right	15 (68%)	3 (75%)	2 (67%)	5 (62%)	2 (50%)	2 (100%)	1 (100%)		12 (67%)	3 (75%)		2 (100%)	2 (40%)	5 (83%)	1 (33%)	5 (83%)		11 (69%)	4 (67%)	
**Gender**																				
Female	11 (50%)	3 (75%)	1 (33%)	4 (50%)	2 (50%)	0 (0%)	1 (100%)	0.6	8 (44%)	3 (75%)	0.6	2 (100%)	2 (40%)	2 (33%)	2 (67%)	3 (50%)	0.7	7 (44%)	4 (67%)	0.6
Male	11 (50%)	1 (25%)	2 (67%)	4 (50%)	2 (50%)	2 (100%)	0 (0%)		10 (56%)	1 (25%)		0 (0%)	3 (60%)	4 (67%)	1 (33%)	3 (50%)		9 (56%)	2 (33%)	
Patient age	64 (56, 68)	58 (55, 62)	49 (48, 59)	64 (62, 66)	64 (55, 72)	78 (77, 80)	67 (67, 67)	0.2	64 (56, 67)	69 (64, 72)	0.2	57 (55, 59)	66 (65, 68)	69 (52, 74)	64 (61, 65)	60 (56, 66)	0.8	64 (58, 67)	62 (54, 70)	>0.9
Hospitalization (days)	9.50 (9.00, 10.75)	10.00 (9.00, 11.25)	9.00 (8.50, 9.00)	10.00 (8.75, 10.00)	11.50 (10.75, 12.00)	8.50 (8.25, 8.75)	8.00 (8.00, 8.00)	0.073	9.00 (9.00, 10.00)	10.50 (9.50, 11.25)	0.5	11.50 (11.25, 11.75)	9.00 (9.00, 9.00)	9.00 (8.00, 10.75)	12.00 (10.50, 12.00)	9.50 (9.00, 10.00)	0.2	9.00 (8.75, 10.00)	11.50 (10.25, 12.00)	0.045
**History of cerebrovascular disease**																				
No	22 (100%)	4 (100%)	3 (100%)	8 (100%)	4 (100%)	2 (100%)	1 (100%)		18 (100%)	4 (100%)		2 (100%)	5 (100%)	6 (100%)	3 (100%)	6 (100%)		16 (100%)	6 (100%)	
Hypertension	12 (55%)	2 (50%)	1 (33%)	5 (62%)	3 (75%)	1 (50%)	0 (0%)	0.9	10 (56%)	2 (50%)	>0.9	0 (0%)	4 (80%)	3 (50%)	1 (33%)	4 (67%)	0.4	9 (56%)	3 (50%)	>0.9
Diabetes	9 (41%)	1 (25%)	2 (67%)	4 (50%)	1 (25%)	1 (50%)	0 (0%)	0.9	8 (44%)	1 (25%)	0.6	0 (0%)	4 (80%)	2 (33%)	0 (0%)	3 (50%)	0.2	8 (50%)	1 (17%)	0.3
Coronary Heart Disease	2 (9.1%)	1 (25%)	0 (0%)	1 (12%)	0 (0%)	0 (0%)	0 (0%)	>0.9	2 (11%)	0 (0%)	>0.9	0 (0%)	1 (20%)	0 (0%)	1 (33%)	0 (0%)	0.4	2 (12%)	0 (0%)	>0.9
Hyperlipidemia	9 (41%)	0 (0%)	2 (67%)	5 (62%)	1 (25%)	1 (50%)	0 (0%)	0.3	8 (44%)	1 (25%)	0.6	0 (0%)	4 (80%)	2 (33%)	0 (0%)	3 (50%)	0.2	8 (50%)	1 (17%)	0.3
**TMS protocol**																				
High freq	10 (45%)	2 (50%)	0 (0%)	4 (50%)	2 (50%)	1 (50%)	1 (100%)	0.7	7 (39%)	3 (75%)	0.3	0 (0%)	2 (40%)	3 (50%)	1 (33%)	4 (67%)	0.5	7 (44%)	3 (50%)	0.7
iTBS	5 (23%)	2 (50%)	1 (33%)	1 (12%)	1 (25%)	0 (0%)	0 (0%)		4 (22%)	1 (25%)		2 (100%)	0 (0%)	1 (17%)	1 (33%)	1 (17%)		3 (19%)	2 (33%)	
Low freq	7 (32%)	0 (0%)	2 (67%)	3 (38%)	1 (25%)	1 (50%)	0 (0%)		7 (39%)	0 (0%)		0 (0%)	3 (60%)	2 (33%)	1 (33%)	1 (17%)		6 (38%)	1 (17%)	
**TMS side**																				
Contralateral	10 (45%)	2 (50%)	2 (67%)	4 (50%)	1 (25%)	1 (50%)	0 (0%)	>0.9	10 (56%)	0 (0%)	0.1	1 (50%)	3 (60%)	2 (33%)	2 (67%)	2 (33%)	0.9	9 (56%)	1 (17%)	0.2
Ipsilateral	12 (55%)	2 (50%)	1 (33%)	4 (50%)	3 (75%)	1 (50%)	1 (100%)		8 (44%)	4 (100%)		1 (50%)	2 (40%)	4 (67%)	1 (33%)	4 (67%)		7 (44%)	5 (83%)	

^a^n (%); median (IQR)

^b^Fisher's exact test; Kruskal–Wallis rank-sum test.

^c^Fisher's exact test; Wilcoxon rank-sum test.

The rTMS treatment targeted the primary motor cortex (M1) in all patients. The targets were at equal proportions of the right (*n* = 11) and left hemispheres (*n* = 11), although varied based on the frequency of rTMS targeting ipsilateral or contralateral to the lesion varied further by rTMS protocol (Table 3). Decisions on which hemisphere rTMS was delivered to relative to the lesion site were made by two independent stroke neurologists based on radiographic findings at patient presentation. The treatment intensity was most commonly of high frequency (*n* = 10, 45%). The type of TMS protocol was not associated with scores at any time point on the NIHSS, BI, or WFMT scales (*p* > 0.05 each). However, the use of iTBS was associated with lower scores on the FMA scale at 1-day (*p* = 0.03) and 30-day (*p* = 0.02) post-stroke.

**Table 3:** Patient demographics by TMS protocol.

**Characteristic**	**High frequency, *N* = 10**	**iTBS, *N* = 5**	**Low frequency, *N* = 7**	***p*-value**
**Lesion side**				
Left	4 (40%)	1 (20%)	2 (29%)	0.9
Right	6 (60%)	4 (80%)	5 (71%)	
**Gender**				
Female	5 (50%)	4 (80%)	2 (29%)	0.3
Male	5 (50%)	1 (20%)	5 (71%)	
Age	66 (64, 70)	53 (52, 58)	65 (56, 68)	0.035
Hospitalization duration (days)	9.00 (9.00, 10.00)	11.00 (10.00, 12.00)	9.00 (8.50, 11.00)	0.3
**History of cerebrovascular disease**				
No	10 (100%)	5 (100%)	7 (100%)	
History of hypertension	8 (80%)	0 (0%)	4 (57%)	0.020
History of diabetes	6 (60%)	1 (20%)	2 (29%)	0.3
History of coronary heart disease	2 (20%)	0 (0%)	0 (0%)	0.5
History of hyperlipidemia	6 (60%)	1 (20%)	2 (29%)	0.3
**TMS side**				
Contralateral	3 (30%)	1 (20%)	6 (86%)	0.041
Ipsilateral	7 (70%)	4 (80%)	1 (14%)	

### 3.1. Functional assessment outcomes

**Figure 1:**
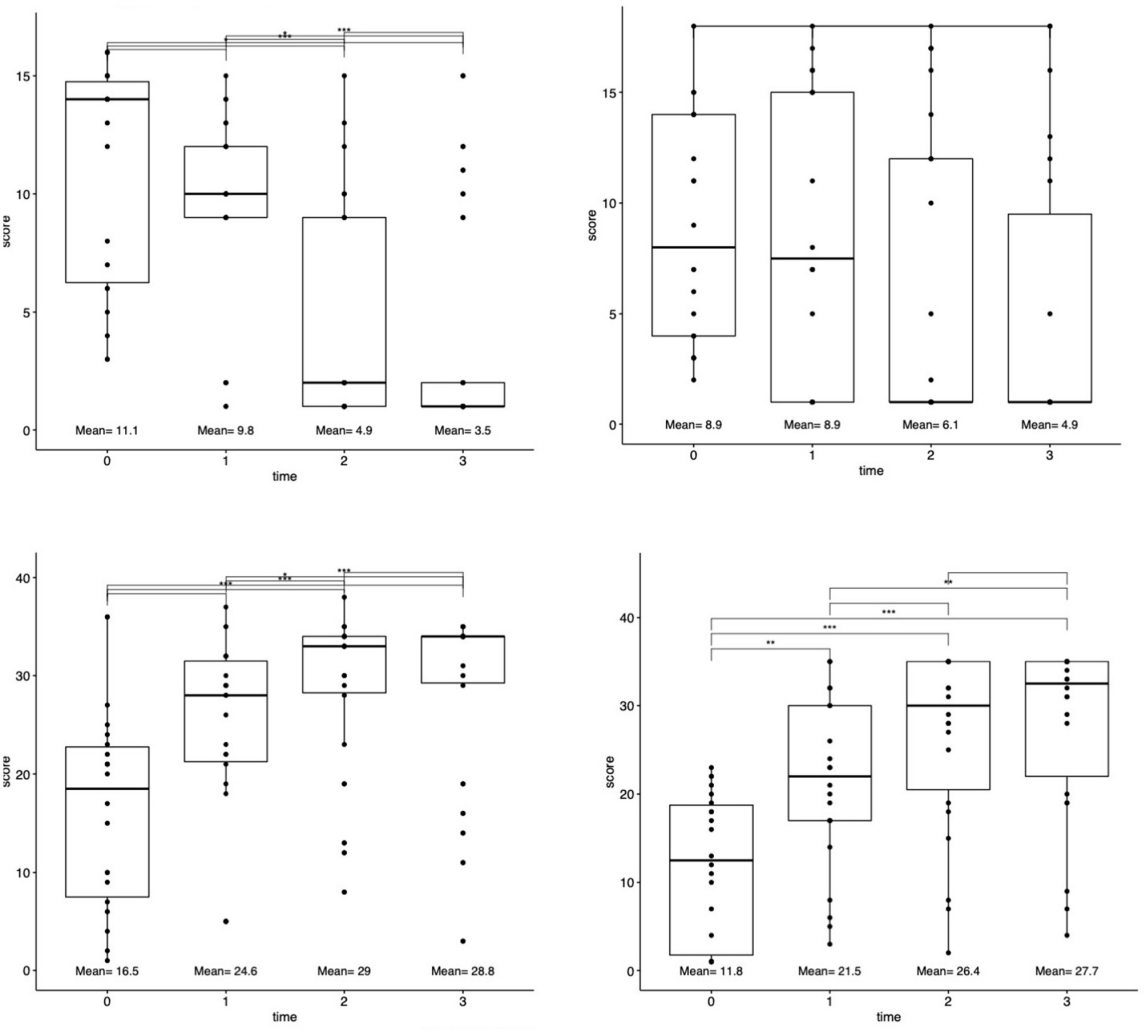
Changes in functional outcomes after rTMS treatment. Patient functional status scores for each scale (NIHSS, FMA, BI, and WMFT) were assessed at four-time points: baseline at presentation, 1-day after rTMS, 30 days after rTMS, and 90 days after rTMS. Top lines connect each time point. ^*^p < 0.05, ^**^p < 0.001, and ^***^p < 0.0001.

Functional outcomes were examined between four standardized stroke scales between four-time points (baseline pre-TMS and 1-day, 30-day, 90-day post-TMS). A significant improvement between all time-points was demonstrated according to the NIHSS (Kendall's *W* = 0.51, large), FMA (Kendall's *W* = 0.59, large), and WFMT (Kendall's *W* = 0.02, small) scales (each *p* < 0.0001). The change in the BI scale was non-significant (*p* = 0.67). Mean values at each time point are presented in Figure 1. *Post hoc* testing demonstrated significant differences between the time points of baseline before TMS and 1-day (*p* = 0.001) as well as 30-day post-TMS (*p* < 0.0001) on the NIHSS scale; significant differences between the time points of baseline before TMS and 30-day (*p* = 0.001) as well as 90-day post-TMS (*p* < 0.001) and also between 1-day post-TMS, 30-day post-TMS (*p* = 0.02), and 90-day (*p* < 0.001) post-TMS on the FMA scale; significant differences between the time points of baseline before TMS and 1-day (*p* = 0.006), 30-day post-TMS (*p* < 0.0001), and 90-day post-TMS (*p* < 0.0001) as well as between 1-day post-TMS and 90-day post-TMS (*p* = 0.002).

### 3.2. Connectivity outcomes

**Figure 2:**
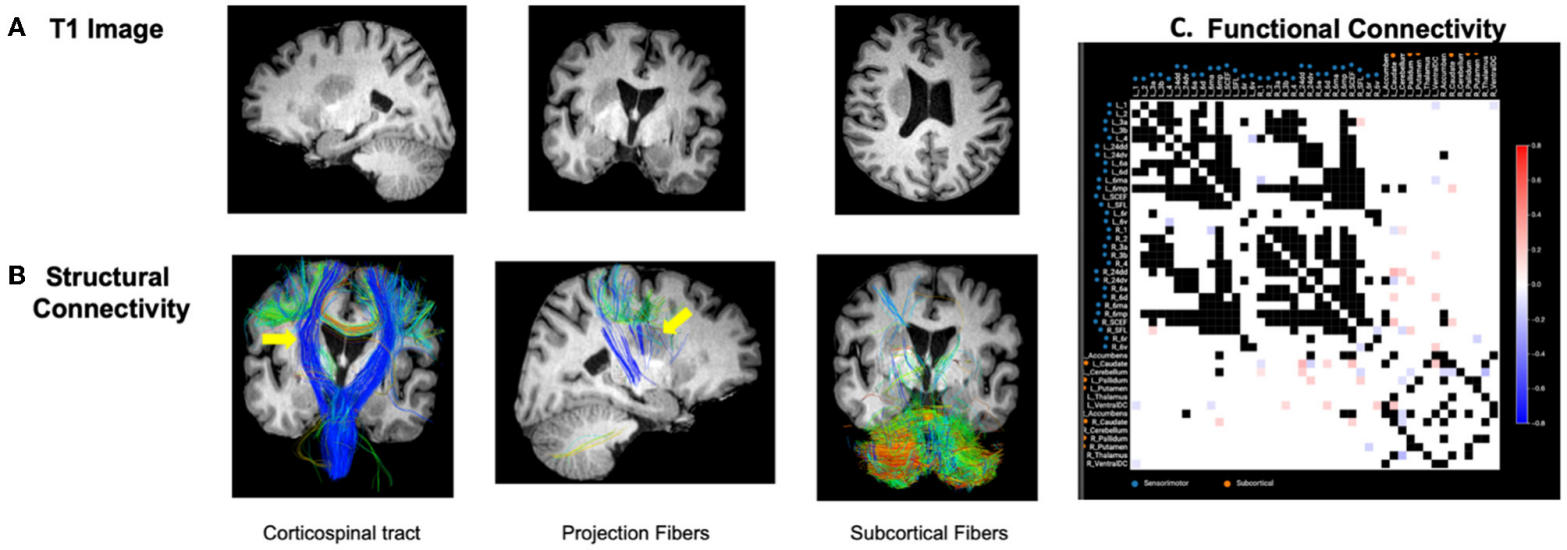
Case example. **(A)** Patient with right-sided stroke presented significant left upper and lower extremity motor deficits. **(B)** Structural tractography revealed the lesion was directly inside the CST and cortical–subcortical projection fibers and an appreciable visual decrease in the integrity of the right CST fibers was identified (represented by yellow arrows). Subcortical fibers were relatively intact from the lesion. **(C)** Functional connectivity revealed a number of hyperconnected (red) and hypoconnected (blue) cortical and subcortical regions compared to the normative functional connectivity of healthy adults. As detailed in the methods, the highest variance 1/3 of pairs were excluded to further reduce the false-discovery rate given these areas may be prone to false discovery due to inter-individual variability in normal subjects. These areas are represented as black in the connectivity matrix. White boxes represent areas within the normative distribution compared to healthy subjects.

Structural and functional connectivities were measured based on individualized connectomic analyses. A case example is presented in Figure 2. These outcomes were addressed below in the next section based on clustering analyses.

### 3.3. Cluster analysis based on standardized stroke scales

**Figure 3:**
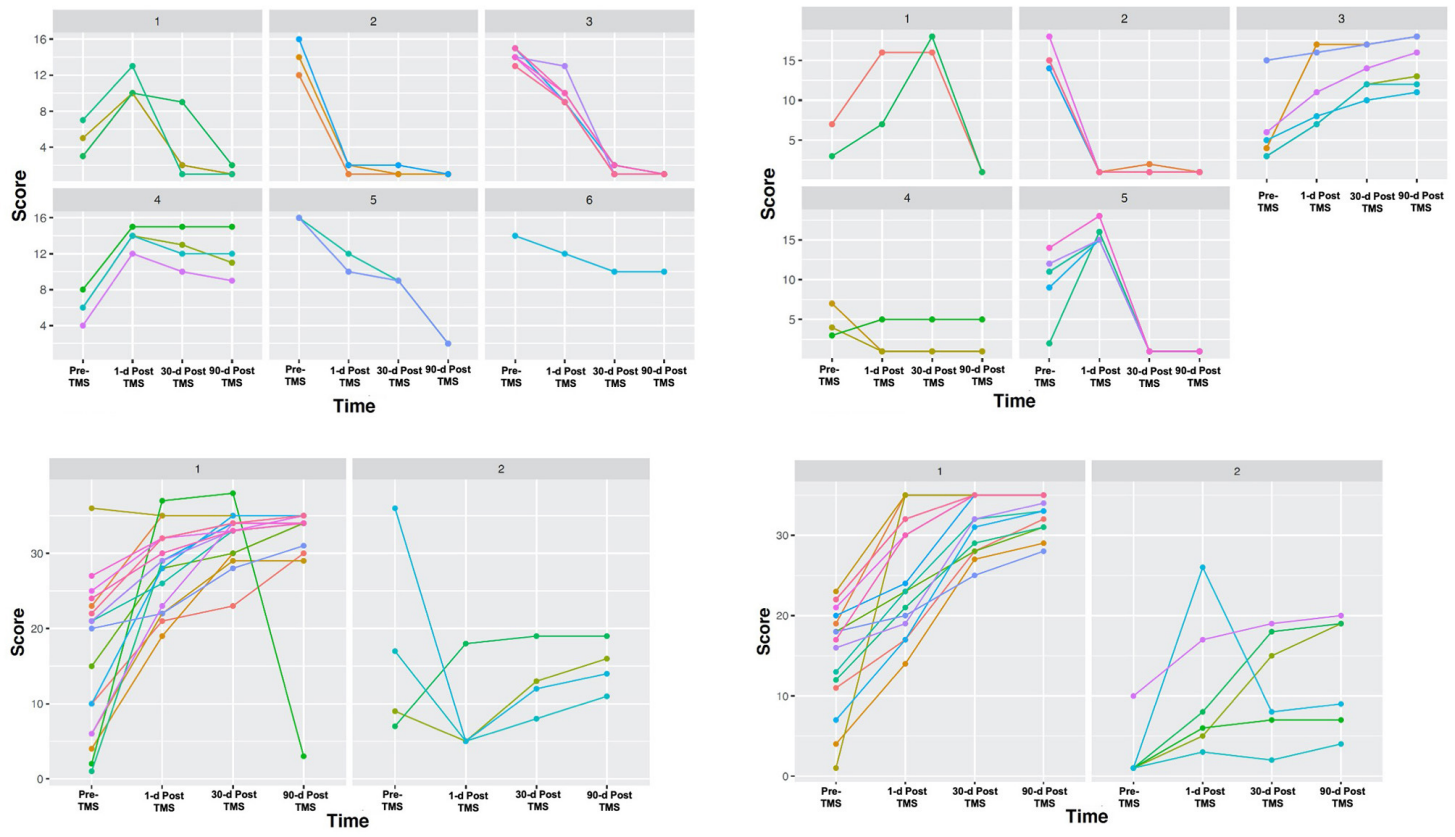
Unique stroke recovery trajectories. Different groups are presented according to cluster analyses using outcomes on the four standardized stroke scales at four-time points. Patient functional status scores were assessed according to: (1) National Institutes of Health Stroke Scale (NIHSS) **(top left)**, (2) Barthel Index (BI) **(top right)**, (3) Fugl-Meyer Assessment (FMA) **(bottom left)**, and (4) Wolf Motor Function Test (WMFT) **(bottom right)**. Each patient score was assessed at four-time points in order to obtain long-term data: (1) at presentation, (2) 1-day after treatment, (3) 30-days after treatment, and (4) 90-days after treatment. While our sample included *n* = 22, individual clusters contained occasional overlapping lines in patients with the same scores. On the NIHSS panel, two patients in cluster 3 had the same score. On the BI panel, two patients in cluster 5 had the same score. On the WMFT, two patients in cluster 1 had the same score.

Cluster analyses based on total scores at four-time points revealed unique clusters, suggesting the presence of different types of patient recovery trajectories in this cohort. These ML-based clustering analyses were completed for each standardized stroke scale (Figure 3). According to the optimal number of unique clusters by the silhouette coefficient, six unique patient trajectories existed for the NIHSS scale, two for the FMA scale, five for the BI scale, and two for the WFMT scale. The silhouette coefficients for each of these scales were 0.59 (NIHSS), 0.52 (FMA), 0.57 (BI), and 0.57 (WFMT). A table comparing patient demographics in the total study sample and by individual clusters is presented in Table 2. There were no significant differences between individual clusters according to individual patient demographics alone except a higher length of hospital duration for cluster 2 compared to cluster 1 on the WFMT scale.

Further inspection of the recovery trajectory profile of each of these scales reveals some important trends. Most importantly, despite some similarities between clusters for each scale (e.g., high- or low-functional status prior to TMS and at the final 90-day time point following TMS), individual clusters varied significantly in terms of whether or not they experienced transient 1- and 30-day declines. These trends in trajectories can be seen in Figure 3. As an example, visually clusters 1 and 4 had similar baseline stroke impairment and 1-day post-TMS scores on the NIHSS scale, but cluster 1 then went on to improve 30 days and 90 days later, while cluster 4 remained the same. Interestingly, while there were no significant differences on the BI scale overall for the cohort, ML-based analyses were able to highlight those patients who did respond (e.g., cluster 3), and how other groups who had similar initial scores to these patients then go on to decline (e.g., clusters 1 and 5).

### 3.4. Connectivity differences between individual clusters

After ML-based analyses were able to identify individual stroke recovery trajectories according to each scale, we next sought to examine differences in structural and functional connectivities between these trajectories. Although some observable trends were noted between clusters on the NIHSS, FMA, and WFMT scales in structural and functional connectivity elements, these visual trends did not reach statistical significance (*p* > 0.05). However, a number of significant differences in structural and functional connectivity changes were identified between clusters on the BI scale. Importantly, these differences prominently differed for the patients who did improve on this scale compared to other clusters. Given our ML-based analyses identified individual trajectories according to each scale regardless of how the overall cohort responded on that specific scale, we focus on connectivity differences for the BI scale below in further detail.

**Figure 4:**
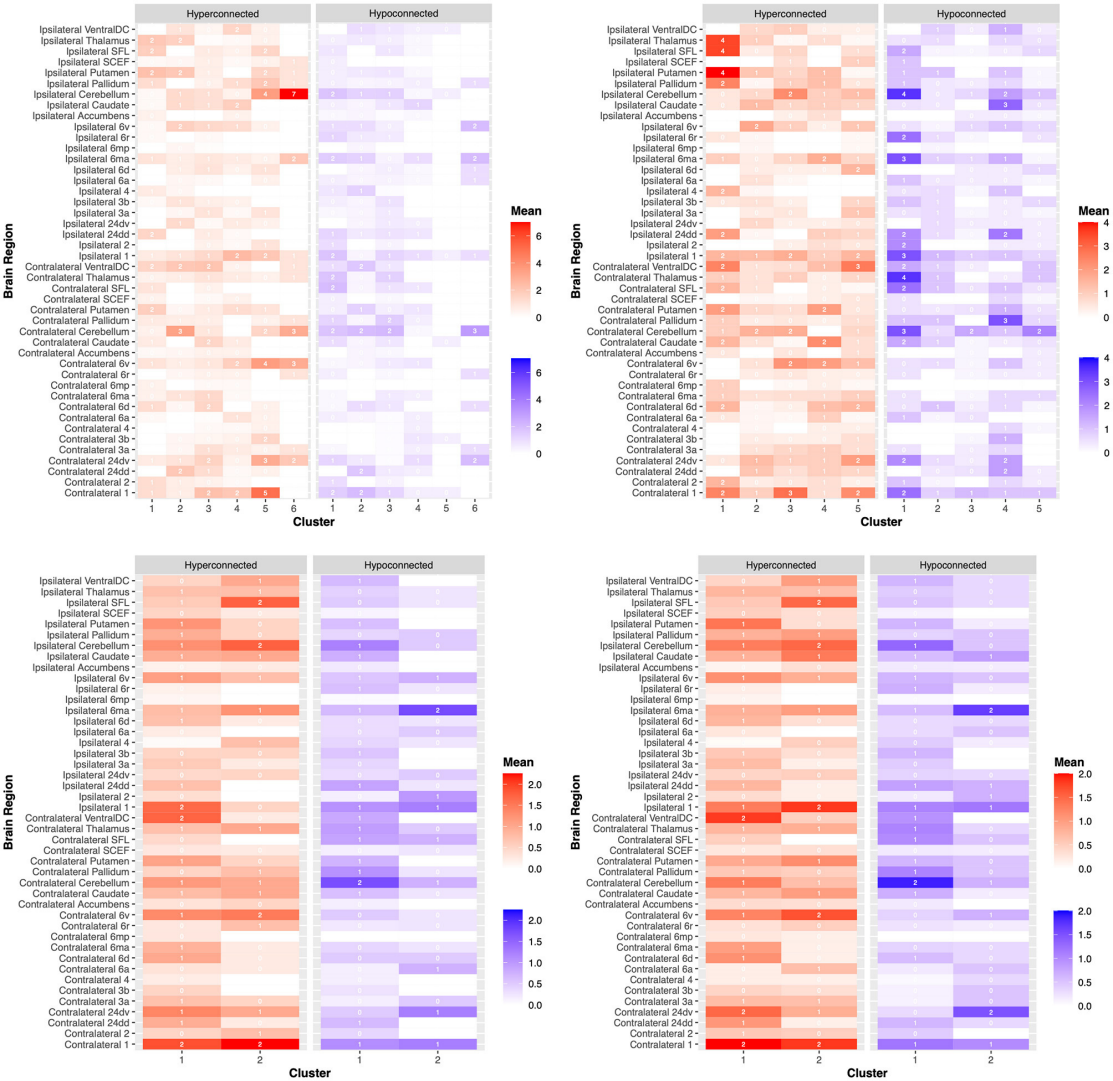
Dysfunctional connectivity between patient clusters. Connectivity anomalies are demonstrated on a heat map between patients according to clustering analyses for the (1) National Institutes of Health Stroke Scale (NIHSS) **(top left)**, (2) Barthel Index (BI) **(top right)**, (3) Fugl-Meyer Assessment (FMA) **(bottom left)**, and (4) Wolf Motor Function Test (WMFT) **(bottom right)**. Hyperconnected parcels are demonstrated in red, with a higher mean number of hyperconnections in dark red and a lower mean number of hyperconnections in light red. Hypoconnected parcels are demonstrated in blue, with a higher mean number of hypoconnections in dark blue and a lower mean number of hypoconnections in light blue. Each brain region, ipsilateral or contralateral to the stroke site, is labeled on the y-axis. Individual patient clusters are on the x-axis. These outcomes are further demonstrated in the Supplementary material.

We provide a heatmap of these connectivity differences for each scale and related clusters in Figure 4 as well as expanded results in the Supplementary material.

#### 3.4.1. Functional connectivity differences between BI clusters

The number of functional connectivity 3-sigma outliers (“anomalies”) between clusters was investigated for both cortical and subcortical connections and the total number of hypoconnected and hyperconnected anomalies.

When investigating specific individual cortical parcels, a number of significant motor regions differed between clusters. Individual groups differed in the mean number of ipsilateral hyperconnected supplementary and cingulate eye field (SCEF) areas of the pre-supplementary motor area (cluster 3 = 0.7 anomalies, cluster 1 = 1 anomaly, no anomalies for other clusters; *p* = 0.04). Although, these differences were not statistically significant between individual clusters on *post hoc* analyses but rather just for all groups together. Similar overall differences were found for SCEF on the ipsilateral side for hypoconnections, where only cluster 1 demonstrated an anomaly (*p* = 0.04). *Post hoc* testing revealed that these ipsilateral hypoconnections were significantly different between group 1 with all other clusters, including clusters 2 (*p* = 0.02), 3 (*p* = 0.02), 4 (*p* = 0.04), and 5 (*p* = 0.04). Differences were also present for the number of hypoconnections with area 24dd contralateral to the lesion side (*p* = 0.02), although *post hoc* analyses revealed differences between individual groups did not reach statistical significance (*p* > 0.05).

When examining subcortical structures, differences mostly existed between groups for subcortical connections which were hypoconnected rather than hyperconnected, specifically with the pallidum, caudate, and thalamus. Significant differences were found for the number of hypoconnections with the contralateral pallidum (cluster 1 = 1.0 anomaly, 2 = 1.0, 3 = 0, 4 = 3.0, 5 = 0.7; *p* = 0.02). *Post hoc* analyses revealed clusters 3 and 4 significantly differed the most (*p* = 0.007). Significant differences were found for the number of hypoconnections with the contralateral thalamus (cluster 1 = 3.5 anomalies, 2 = 1.0, 3 = 0, 4 = 0.3, 5 = 1.0; *p* = 0.02). *Post hoc* analyses revealed that clusters 1 and 3 significantly differed the most (*p* = 0.05). Significant differences were found for the number of hypoconnections with the ipsilateral caudate (cluster 1 = 0.5 anomalies, 2 = 0.4, 3 = 0, 4 = 2.7, 5 = 0.5; *p* = 0.02). *Post hoc* analyses revealed that clusters 3 and 4 significantly differed the most (*p* = 0.02).

The mean number of contralateral cortical parcels which were hypoconnected differed between clusters (cluster 1 = 12 anomalies, 2 = 4.4, 3 = 3.8, 4 = 10, 5 = 4.2; *p* = 0.05). The mean number of hypoconnected ipsilateral cortical parcels between clusters followed a similar trend but did not reach statistical significance (cluster 1 = 19 anomalies, 2 = 6.6, 3 = 4.0, 4 = 9.0, 5 = 5.3; *p* = 0.09).

Differences between other individual parcellations are demonstrated in Figure 4 and in the Supplementary material which did not reach statistical significance.

#### 3.4.2. Structural connectivity differences between BI clusters

Differences in the visual appearance and lesion proximity of different clusters were examined given the importance of white matter integrity in post-stroke outcomes and treatment responses (47–49). When examining the proximity of the lesion to white matter fibers on DTI, there was a significant difference between groups for cortical–subcortical projection fibers (*p* = 0.03), but not for subcortical fibers (*p* = 0.71) or the CST (*p* = 0.68). For cortical–subcortical projection fibers, proximity was significantly different between clusters (*p* = 0.033). Proximity was not a predictor of 90-day BI score alone (*p* > 0.05). Similarly, when examining the disruption of white matter fibers on DTI, there was a significant difference between groups regarding the visual integrity of cortical–subcortical projection fibers (*p* = 0.04), but not for subcortical fibers (*p* = 0.52) or the CST (*p* = 0.38). For cortical–subcortical projection fibers, visual integrity was significantly different between clusters (*p* = 0.047). Visual integrity was not a predictor of 90-day BI score alone (*p* > 0.05).

## 4. Discussion

Despite a clear understanding that stroke patients vary significantly in regard to their recovery trajectory, there remains a poor understanding of how to gain further insight into this process during motor recovery treatment. Many scales which assess patient functional outcomes (motor, sensory, and cognitive) have been developed to predict individual stroke recovery in order to guide treatment decisions; however, these scales remain heterogenous and there is little consensus on their clinical value across the field (50). In this study, a novel approach was taken to identify different recovery phenotypes following rTMS treatment for acute stroke patients and specifically with unique insight from personalized connectomic information. Namely, a reverse approach was taken which clustered patients with machine learning analyses according to baseline and post-rTMS functional scores on validated stroke scales, rather than just grouping patients according to clinical presentation characteristics alone (45). While we found significant improvements in functional recovery for patients from baseline up to 90-day post-rTMS treatment across our entire sample, evidence was found for clusters of specific patients with distinct recovery trajectories. Furthermore, these treatment response phenotypes could partially be differentiated according to their unique structural and functional connectivity disruptions in the motor network despite all suffering from “similar” acute strokes.

In many controlled trials, stroke patients are largely treated as if they have the same underlying problem, despite it being known that there are unique neurobiological differences between patients (19). Thus, it is unsurprising to find that there have been many conflicting results in functional outcomes for similar stroke treatments, such as TMS, across different trials (1, 14). What is interesting in the current study is that despite not being a largely powered study, a number of quantitative differences were found existing in structural and functional connectivity between individuals and this information could differentiate unique phenotypes in rTMS treatment responses and recovery on a standardized stroke scale. Thus, functional and structural connectivity analyses may allow for additional assistance in determining the prognosis of the patient as well as for trial designs in more appreciable ways at the single subject level than many other predicting tools which do not account for neurobiological differences between individuals (51).

Spontaneous stroke recovery in functional ability, such as motor functions, has been reiteratively demonstrated to be dependent on underlying brain network damage and the network's capacity for functional re-organization (19, 24–26). Based on our study, different phenotypes according to the Barthel scale varied in their total number of abnormal functional connections to cortical parcellations. The connectivity of these parcellations in the sensorimotor network has been well-described previously (52, 53) and are well-known regions involved in motor functioning (54). In particular, the mean total of hypoconnected parcels contralateral to the lesion side differed between specific trajectories. Similar results have been found in previous study with less anatomic specificity (55, 56), although early identification of the specific contralateral hypoconnected sensorimotor connections which can be normalized with neuromodulatory treatments is important for facilitating clinical improvements in the functional activity and motor impairments (48). Furthermore, significant abnormalities included dysfunctional connectivity of ipsilateral pre-supplementary motor (pre-SMA) areas, ipsilateral caudate connections, and contralateral pallidum connections. As an example, patients in Barthel clusters 1 and 2 were similar in their lower long-term 90-day scores but differed in their trajectory such that cluster 1 had a transient improvement at 30 days before declining in function. Simultaneously, cluster 1 had a greater number of hypoconnected ipsilateral connections to the supplementary and cingulate eye field (SCEF) of the pre-SMA. SCEF is a motor planning and initiation area believed to be a likely a major point of informational outflow from higher-order networks into the motor system due to shared network affiliation (57), and damage to its connections may be a major cause of problems with the initiation of goal-directed behaviors, such as in SMA syndrome (58–60). Another example can be seen with clusters 3 and 4 which had similar low Barthel starting points but varied in their long-term scores (high vs. low). Cluster 4 had high functional scores at 90 days, and also had a greater number of abnormally decreased connections with the ipsilateral caudate and contralateral pallidum compared to cluster 4. Damage to each of these structures has been extensively correlated with a variety of functional deficits (48, 61), and therefore, identifying these functional connections may provide important connectomic features to model stroke severity and recovery moving forward.

In addition to the insight provided by functional connectivity, structural connectivity analyses have also been suggested to provide additional information to better understand stroke recovery (19, 62, 63). In the current study, individual clusters on the Barthel scale were significantly different in regard to their projection fiber integrity. Projection fibers are white matter connections that link cortical and subcortical structures and facilitate a variety of motor and non-motor functions. Although stroke studies incorporating structural connectivity analyses focus on the CST and its connections in the motor network (64), projection fibers are also extensively damaged in stroke patients and are important in understanding post-stroke deficits despite not being extensively studied to date (47). In our sample, the integrity of these fibers alone was not predictor of post-TMS scores; although this is not entirely surprising given, these connectomic elements are just one important structure that likely contributes to overall function and recovery ability. Tools may be created which can model the severity of white matter integrity of projection fibers in addition to the CST and other white matter connections (e.g., commissural fibers) to better understand motor impairment (47), but additional studies should also examine their non-motor correlates post-stroke. By mapping this lesion topography to white matter connections, structural anatomic correlates can be identified for overall stroke severity and post-stroke outcomes which may aid in decisions for early rehabilitation strategies tailored to specific patients but also perhaps for individual symptoms in future studies (11, 48, 65).

An increase in the number of studies has attempted to incorporate structural–functional analyses to predict motor recovery following stroke. These studies have mainly focused on the CST in relation to predicting motor impairment with variable outcomes (66–69), and have also suggested the volume of the acute lesion (70) may be less important to motor recovery compared to the actual lesion location (71) and integrity of specific underlying white matter bundles (19, 72). These observations highlight one of the main benefits of our analyses, namely the utilization of an anatomically fine surface-based, multi-modal parcellation scheme published by the Human Connectome Project. Parcel-guided analyses may improve our ability to better analyze underlying pathophysiological mechanisms and communicate more anatomically fine results between studies for hypothesis generation (18). Furthermore, parcel-guided treatments can provide us a step forward to more accurate therapeutic targeting (9–11, 73). The efficacy of rTMS treatment is highly dependent on the target location, which can be incorrectly estimated with standard craniometric measurements that often underestimate the localization of underlying structures that often only have millimeter differences across the human scalp (74). While parcel-guided TMS was not utilized in the current study, and rather only to analyze and report our data, this study provides an example of the feasibility and importance of such specific analyses which should be examined further in future study for the clinical relevance of such analyses.

The current study sought to use machine learning to identify unique patient trajectories following acute stroke and then to examine how connectivity information may provide additional insight into these differences. While accomplishing this goal in this current study, it is important to note that the current study did not attempt to examine the intricacies and mechanisms of TMS treatment or associated patient responses. It is well-known that differences in TMS parameters may affect patient responses (75, 76), but this was not examined in the current study and instead, our results may at most in this context point to the need to identify precise anatomic neuromodulatory targets, but not the efficacy in targeting these regions. Furthermore, an obvious point brought out by our analyses is how stroke patients may have unique recovery trajectories but also that these trajectories may vary between different scales such that a select group of patients “responding” on one scale may or may not be a responder on a different scale. Although not the focus of study in the current work a large body of research has also attempted to look at these differences which presents an important area of research moving forwards which connectomics may also provide valuable information (77). Nonetheless, our results instead highlight the ability of ML-based analyses to identify and highlight trajectories irrespective of a responder or non-responder status, and then how connectomic features can differentiate some of these patients, as seen with the Barthel Index.

Our study included a small sample size of patients from a single institution. Thus, while individualized connectivity analyses produced a large amount of data for each single patient, these biases could have influenced our statistical analyses and therefore although connectivity differences may have existed between clusters on other scales, these differences may not have been identified in the current dataset. Our methods utilized a unique way to investigate functional connectivity analyses using connectivity “anomalies.” Given small changes in functional connectivity can be difficult and too vague to interpret, our use of 3-sigma anomalies provides a novel way to highlight likely meaningful changes in a patients connectome in response to pathology or intervention; however, our structural connectivity-based analyses relied on the visual inspection of DTI as other have completed (39) and therefore may have been subject to additional bias. Structural connectivity provides a meaningful way to examine major differences in a patient's white matter bundles and identify gross patterns between individuals, but when examined alone without additional information these data should not be over-interpreted. In light of these limitations, future studies with larger datasets and additional statistical power should look to examine individual scale subcomponents with greater statistical certainty as it relates to precise connectivity features (65). This is an important area of future research as we transition toward a period where technology now exists for highly specialized targeting according to individual deficits (9, 11, 73).

Despite having limited power, a number of quantitative differences in structural and functional connectivity were identified which could differentiate unique patient recovery trajectories on a standardized stroke scale and provide insight into their treatment response. A larger sample size may have allowed us to more confidently identify more specific individual parcellations for each cluster and among varying scales. Instead, the current results demonstrate the value of including additional connectomic information on individual patients that may have unique pathophysiological profiles despite similar injuries in order to appropriately guide clinical decision-making and understand treatment capabilities moving forward.

## 5. Conclusion

This study demonstrates the ability to identify unique patient rTMS recovery trajectories between patients and how functional and structural connectivity features can provide additional information in this context. Additional personalized connectivity analyses may allow for an improved understanding of the patient's disease burden or estimate their trajectory and capability for neuromodulatory treatments and therefore represents an important area for future study in larger prospective studies.

## Data availability statement

The original contributions presented in the study are included in the article/Supplementary material, further inquiries can be directed to the corresponding author.

## Ethics statement

The studies involving human participants were reviewed and approved by First Affiliated Hospital of Hainan Medical University Ethics Committee. Written informed consent for participation was not required for this study in accordance with the national legislation and the institutional requirements.

## Author contributions

RC: conceptualization, methodology, original draft, and writing—review and editing. ND and BC: writing—formal analysis, visualization, original draft, and writing—review and editing. XZ: project administration and supervision. XH: project administration, supervision, and writing—review and editing. MS: conceptualization, methodology, and writing—review and editing. LS: rehabilitation function assessment. XW: imaging data acquisition. YL: clinical data collection and analysis. All authors contributed to the article and approved the submitted version.
