# Supplementary material for: Connectomic insight into unique stroke patient recovery after rTMS treatment
Source: Front Neurol. 2023 Jul 6;14:1063408. doi: 10.3389/fneur.2023.1063408 (PMC10359072; doi:10.3389/fneur.2023.1063408)
Supplement: Supplementary file 3 [file Data_Sheet_1.PDF]

numeric outcomes 90 days (1)

	cluster	mean	variable.name...number	scale	univariate.test.name	univariate.p		Key	
1	1	1.25	Red-positiveL_1...3	nihss	kruskal	0.253902753309959		**Variable.name..number**	
2	2	0.666666666666667	Red-positiveL_1...3	nihss	kruskal	0.253902753309959		1) connectivity type	red positive = hyperconnected
3	3	2.25	Red-positiveL_1...3	nihss	kruskal	0.253902753309959			blue negative = hypo connected
4	4	2	Red-positiveL_1...3	nihss	kruskal	0.253902753309959		2) Location	L = contralateral
5	5	5	Red-positiveL_1...3	nihss	kruskal	0.253902753309959			R = ipsilateral
6	6	0	Red-positiveL_1...3	nihss	kruskal	0.253902753309959			
7	1	1.25	Red-positiveL_2...4	nihss	kruskal	0.429181502521092			
8	2	0.666666666666667	Red-positiveL_2...4	nihss	kruskal	0.429181502521092			
9	3	0.5	Red-positiveL_2...4	nihss	kruskal	0.429181502521092			
10	4	0.25	Red-positiveL_2...4	nihss	kruskal	0.429181502521092			
11	5	0	Red-positiveL_2...4	nihss	kruskal	0.429181502521092			
12	6	0	Red-positiveL_2...4	nihss	kruskal	0.429181502521092			
13	1	0	Red-positiveL_3a...5	nihss	kruskal	0.525921880563112			
14	2	0.333333333333333	Red-positiveL_3a...5	nihss	kruskal	0.525921880563112			
15	3	1.125	Red-positiveL_3a...5	nihss	kruskal	0.525921880563112			
16	4	0.75	Red-positiveL_3a...5	nihss	kruskal	0.525921880563112			
17	5	0.5	Red-positiveL_3a...5	nihss	kruskal	0.525921880563112			
18	6	1	Red-positiveL_3a...5	nihss	kruskal	0.525921880563112			
19	1	0	Red-positiveL_3b...6	nihss	kruskal	0.172494857195262			
20	2	0.333333333333333	Red-positiveL_3b...6	nihss	kruskal	0.172494857195262			
21	3	0.375	Red-positiveL_3b...6	nihss	kruskal	0.172494857195262			
22	4	0.5	Red-positiveL_3b...6	nihss	kruskal	0.172494857195262			
23	5	1.5	Red-positiveL_3b...6	nihss	kruskal	0.172494857195262			
24	6	0	Red-positiveL_3b...6	nihss	kruskal	0.172494857195262			
25	1	0	Red-positiveL_4...7	nihss	kruskal	0.645559545450256			
26	2	0	Red-positiveL_4...7	nihss	kruskal	0.645559545450256			
27	3	0.25	Red-positiveL_4...7	nihss	kruskal	0.645559545450256			
28	4	0.25	Red-positiveL_4...7	nihss	kruskal	0.645559545450256			
29	5	0.5	Red-positiveL_4...7	nihss	kruskal	0.645559545450256			
30	6	0	Red-positiveL_4...7	nihss	kruskal	0.645559545450256			
31	1	0	Red-positiveL_24dd...8	nihss	kruskal	0.129342003740699			
32	2	2	Red-positiveL_24dd...8	nihss	kruskal	0.129342003740699			
33	3	1.25	Red-positiveL_24dd...8	nihss	kruskal	0.129342003740699			
34	4	0.25	Red-positiveL_24dd...8	nihss	kruskal	0.129342003740699			
35	5	0.5	Red-positiveL_24dd...8	nihss	kruskal	0.129342003740699			
36	6	0	Red-positiveL_24dd...8	nihss	kruskal	0.129342003740699			
37	1	0.75	Red-positiveL_24dv...9	nihss	kruskal	0.483308279527692			
38	2	1	Red-positiveL_24dv...9	nihss	kruskal	0.483308279527692			
39	3	1.625	Red-positiveL_24dv...9	nihss	kruskal	0.483308279527692			
40	4	0.5	Red-positiveL_24dv...9	nihss	kruskal	0.483308279527692			
41	5	3	Red-positiveL_24dv...9	nihss	kruskal	0.483308279527692			
42	6	2	Red-positiveL_24dv...9	nihss	kruskal	0.483308279527692			
43	1	0.25	Red-positiveL_6a...10	nihss	kruskal	0.505355012590452			
44	2	0	Red-positiveL_6a...10	nihss	kruskal	0.505355012590452			
45	3	0.125	Red-positiveL_6a...10	nihss	kruskal	0.505355012590452			
46	4	1	Red-positiveL_6a...10	nihss	kruskal	0.505355012590452			
47	5	0.5	Red-positiveL_6a...10	nihss	kruskal	0.505355012590452			
48	6	0	Red-positiveL_6a...10	nihss	kruskal	0.505355012590452			
49	1	1.25	Red-positiveL_6d...11	nihss	kruskal	0.138236896912128			
50	2	0.333333333333333	Red-positiveL_6d...11	nihss	kruskal	0.138236896912128			
51	3	1.5	Red-positiveL_6d...11	nihss	kruskal	0.138236896912128			
52	4	0	Red-positiveL_6d...11	nihss	kruskal	0.138236896912128			
53	5	0.5	Red-positiveL_6d...11	nihss	kruskal	0.138236896912128			
54	6	0	Red-positiveL_6d...11	nihss	kruskal	0.138236896912128			
55	1	0.5	Red-positiveL_6ma...12	nihss	kruskal	0.321387526876191			
56	2	1	Red-positiveL_6ma...12	nihss	kruskal	0.321387526876191			
57	3	1.375	Red-positiveL_6ma...12	nihss	kruskal	0.321387526876191			
58	4	0.25	Red-positiveL_6ma...12	nihss	kruskal	0.321387526876191			

59	5	0	Red-positiveL_6ma...12	nihss	kruskal	0.321387526876191			
60	6	0	Red-positiveL_6ma...12	nihss	kruskal	0.321387526876191			
61	1	0.5	Red-positiveL_6mp...13	nihss	kruskal	0.879148461703196			
62	2	0	Red-positiveL_6mp...13	nihss	kruskal	0.879148461703196			
63	3	0.25	Red-positiveL_6mp...13	nihss	kruskal	0.879148461703196			
64	4	0.25	Red-positiveL_6mp...13	nihss	kruskal	0.879148461703196			
65	5	0	Red-positiveL_6mp...13	nihss	kruskal	0.879148461703196			
66	6	0	Red-positiveL_6mp...13	nihss	kruskal	0.879148461703196			
67	1	0.5	Red-positiveL_SCEF...14	nihss	kruskal	0.682004800192683			
68	2	0	Red-positiveL_SCEF...14	nihss	kruskal	0.682004800192683			
69	3	0.25	Red-positiveL_SCEF...14	nihss	kruskal	0.682004800192683			
70	4	0.5	Red-positiveL_SCEF...14	nihss	kruskal	0.682004800192683			
71	5	0	Red-positiveL_SCEF...14	nihss	kruskal	0.682004800192683			
72	6	0	Red-positiveL_SCEF...14	nihss	kruskal	0.682004800192683			
73	1	1	Red-positiveL_SFL...15	nihss	kruskal	0.427117766800729			
74	2	0	Red-positiveL_SFL...15	nihss	kruskal	0.427117766800729			
75	3	0.5	Red-positiveL_SFL...15	nihss	kruskal	0.427117766800729			
76	4	0	Red-positiveL_SFL...15	nihss	kruskal	0.427117766800729			
77	5	0	Red-positiveL_SFL...15	nihss	kruskal	0.427117766800729			
78	6	0	Red-positiveL_SFL...15	nihss	kruskal	0.427117766800729			
79	1	0	Red-positiveL_6r...16	nihss	kruskal	0.318572639286791			
80	2	0.333333333333333	Red-positiveL_6r...16	nihss	kruskal	0.318572639286791			
81	3	0.5	Red-positiveL_6r...16	nihss	kruskal	0.318572639286791			
82	4	0.5	Red-positiveL_6r...16	nihss	kruskal	0.318572639286791			
83	5	0	Red-positiveL_6r...16	nihss	kruskal	0.318572639286791			
84	6	1	Red-positiveL_6r...16	nihss	kruskal	0.318572639286791			
85	1	1	Red-positiveL_6v...17	nihss	kruskal	0.3129459873802			
86	2	0.666666666666667	Red-positiveL_6v...17	nihss	kruskal	0.3129459873802			
87	3	0.875	Red-positiveL_6v...17	nihss	kruskal	0.3129459873802			
88	4	1.75	Red-positiveL_6v...17	nihss	kruskal	0.3129459873802			
89	5	3.5	Red-positiveL_6v...17	nihss	kruskal	0.3129459873802			
90	6	3	Red-positiveL_6v...17	nihss	kruskal	0.3129459873802			
91	1	0.75	Red-positiveR_1...18	nihss	kruskal	0.737466050832377			
92	2	1	Red-positiveR_1...18	nihss	kruskal	0.737466050832377			
93	3	1.375	Red-positiveR_1...18	nihss	kruskal	0.737466050832377			
94	4	2.5	Red-positiveR_1...18	nihss	kruskal	0.737466050832377			
95	5	2	Red-positiveR_1...18	nihss	kruskal	0.737466050832377			
96	6	1	Red-positiveR_1...18	nihss	kruskal	0.737466050832377			
97	1	0	Red-positiveR_2...19	nihss	kruskal	0.626116679207013			
98	2	0	Red-positiveR_2...19	nihss	kruskal	0.626116679207013			
99	3	0.5	Red-positiveR_2...19	nihss	kruskal	0.626116679207013			
100	4	0.25	Red-positiveR_2...19	nihss	kruskal	0.626116679207013			
101	5	1	Red-positiveR_2...19	nihss	kruskal	0.626116679207013			
102	6	0	Red-positiveR_2...19	nihss	kruskal	0.626116679207013			
103	1	0	Red-positiveR_3a...20	nihss	kruskal	0.696058704992172			
104	2	0.333333333333333	Red-positiveR_3a...20	nihss	kruskal	0.696058704992172			
105	3	1	Red-positiveR_3a...20	nihss	kruskal	0.696058704992172			
106	4	0.25	Red-positiveR_3a...20	nihss	kruskal	0.696058704992172			
107	5	1	Red-positiveR_3a...20	nihss	kruskal	0.696058704992172			
108	6	0	Red-positiveR_3a...20	nihss	kruskal	0.696058704992172			
109	1	0.25	Red-positiveR_3b...21	nihss	kruskal	0.866905906465408			
110	2	1	Red-positiveR_3b...21	nihss	kruskal	0.866905906465408			
111	3	0.625	Red-positiveR_3b...21	nihss	kruskal	0.866905906465408			
112	4	0.5	Red-positiveR_3b...21	nihss	kruskal	0.866905906465408			
113	5	0	Red-positiveR_3b...21	nihss	kruskal	0.866905906465408			
114	6	0	Red-positiveR_3b...21	nihss	kruskal	0.866905906465408			
115	1	0.75	Red-positiveR_4...22	nihss	kruskal	0.472747918718027			
116	2	0.333333333333333	Red-positiveR_4...22	nihss	kruskal	0.472747918718027			
117	3	0	Red-positiveR_4...22	nihss	kruskal	0.472747918718027			
118	4	0	Red-positiveR_4...22	nihss	kruskal	0.472747918718027			

119	5	0	Red-positiveR_4...22	nihss	kruskal	0.472747918718027			
120	6	0	Red-positiveR_4...22	nihss	kruskal	0.472747918718027			
121	1	1.5	Red-positiveR_24dd...23	nihss	kruskal	0.545925998458994			
122	2	0	Red-positiveR_24dd...23	nihss	kruskal	0.545925998458994			
123	3	0.75	Red-positiveR_24dd...23	nihss	kruskal	0.545925998458994			
124	4	0.25	Red-positiveR_24dd...23	nihss	kruskal	0.545925998458994			
125	5	0	Red-positiveR_24dd...23	nihss	kruskal	0.545925998458994			
126	6	0	Red-positiveR_24dd...23	nihss	kruskal	0.545925998458994			
127	1	0	Red-positiveR_24dv...24	nihss	kruskal	0.209375517935362			
128	2	1.33333333333333	Red-positiveR_24dv...24	nihss	kruskal	0.209375517935362			
129	3	0.25	Red-positiveR_24dv...24	nihss	kruskal	0.209375517935362			
130	4	0.75	Red-positiveR_24dv...24	nihss	kruskal	0.209375517935362			
131	5	0.5	Red-positiveR_24dv...24	nihss	kruskal	0.209375517935362			
132	6	0	Red-positiveR_24dv...24	nihss	kruskal	0.209375517935362			
133	1	0	Red-positiveR_6a...25	nihss	kruskal	0.565521721652305			
134	2	0.333333333333333	Red-positiveR_6a...25	nihss	kruskal	0.565521721652305			
135	3	0.25	Red-positiveR_6a...25	nihss	kruskal	0.565521721652305			
136	4	0	Red-positiveR_6a...25	nihss	kruskal	0.565521721652305			
137	5	0.5	Red-positiveR_6a...25	nihss	kruskal	0.565521721652305			
138	6	0	Red-positiveR_6a...25	nihss	kruskal	0.565521721652305			
139	1	0	Red-positiveR_6d...26	nihss	kruskal	0.516113914362128			
140	2	0.666666666666667	Red-positiveR_6d...26	nihss	kruskal	0.516113914362128			
141	3	1	Red-positiveR_6d...26	nihss	kruskal	0.516113914362128			
142	4	0.5	Red-positiveR_6d...26	nihss	kruskal	0.516113914362128			
143	5	1	Red-positiveR_6d...26	nihss	kruskal	0.516113914362128			
144	6	0	Red-positiveR_6d...26	nihss	kruskal	0.516113914362128			
145	1	1	Red-positiveR_6ma...27	nihss	kruskal	0.595593343760043			
146	2	0.666666666666667	Red-positiveR_6ma...27	nihss	kruskal	0.595593343760043			
147	3	0.875	Red-positiveR_6ma...27	nihss	kruskal	0.595593343760043			
148	4	0.75	Red-positiveR_6ma...27	nihss	kruskal	0.595593343760043			
149	5	0.5	Red-positiveR_6ma...27	nihss	kruskal	0.595593343760043			
150	6	2	Red-positiveR_6ma...27	nihss	kruskal	0.595593343760043			
151	1	0	Red-positiveR_6mp...28	nihss	kruskal	0.670143550784473			
152	2	0.333333333333333	Red-positiveR_6mp...28	nihss	kruskal	0.670143550784473			
153	3	0.125	Red-positiveR_6mp...28	nihss	kruskal	0.670143550784473			
154	4	0	Red-positiveR_6mp...28	nihss	kruskal	0.670143550784473			
155	5	0	Red-positiveR_6mp...28	nihss	kruskal	0.670143550784473			
156	6	0	Red-positiveR_6mp...28	nihss	kruskal	0.670143550784473			
157	1	0.25	Red-positiveR_SCEF...29	nihss	kruskal	0.845131023845981			
158	2	0.333333333333333	Red-positiveR_SCEF...29	nihss	kruskal	0.845131023845981			
159	3	0.625	Red-positiveR_SCEF...29	nihss	kruskal	0.845131023845981			
160	4	0.25	Red-positiveR_SCEF...29	nihss	kruskal	0.845131023845981			
161	5	0.5	Red-positiveR_SCEF...29	nihss	kruskal	0.845131023845981			
162	6	1	Red-positiveR_SCEF...29	nihss	kruskal	0.845131023845981			
163	1	1.75	Red-positiveR_SFL...30	nihss	kruskal	0.783253610056517			
164	2	0	Red-positiveR_SFL...30	nihss	kruskal	0.783253610056517			
165	3	0.75	Red-positiveR_SFL...30	nihss	kruskal	0.783253610056517			
166	4	0.5	Red-positiveR_SFL...30	nihss	kruskal	0.783253610056517			
167	5	1.5	Red-positiveR_SFL...30	nihss	kruskal	0.783253610056517			
168	6	0	Red-positiveR_SFL...30	nihss	kruskal	0.783253610056517			
169	1	0.25	Red-positiveR_6r...31	nihss	kruskal	0.736443059876738			
170	2	0	Red-positiveR_6r...31	nihss	kruskal	0.736443059876738			
171	3	0.25	Red-positiveR_6r...31	nihss	kruskal	0.736443059876738			
172	4	0	Red-positiveR_6r...31	nihss	kruskal	0.736443059876738			
173	5	0	Red-positiveR_6r...31	nihss	kruskal	0.736443059876738			
174	6	0	Red-positiveR_6r...31	nihss	kruskal	0.736443059876738			
175	1	0.25	Red-positiveR_6v...32	nihss	kruskal	0.600634273353754			
176	2	1.66666666666667	Red-positiveR_6v...32	nihss	kruskal	0.600634273353754			
177	3	1.375	Red-positiveR_6v...32	nihss	kruskal	0.600634273353754			
178	4	1.25	Red-positiveR_6v...32	nihss	kruskal	0.600634273353754			

179	5	0.5	Red-positiveR_6v...32	nihss	kruskal	0.600634273353754			
180	6	0	Red-positiveR_6v...32	nihss	kruskal	0.600634273353754			
181	1	0.25	Red-positiveL_Accumber	nihss	kruskal	0.961434194221841			
182	2	0.333333333333333	Red-positiveL_Accumber	nihss	kruskal	0.961434194221841			
183	3	0.5	Red-positiveL_Accumber	nihss	kruskal	0.961434194221841			
184	4	0.5	Red-positiveL_Accumber	nihss	kruskal	0.961434194221841			
185	5	0	Red-positiveL_Accumber	nihss	kruskal	0.961434194221841			
186	6	0	Red-positiveL_Accumber	nihss	kruskal	0.961434194221841			
187	1	0.75	Red-positiveL_Caudate..	nihss	kruskal	0.661328982504582			
188	2	0	Red-positiveL_Caudate..	nihss	kruskal	0.661328982504582			
189	3	1.5	Red-positiveL_Caudate..	nihss	kruskal	0.661328982504582			
190	4	0.75	Red-positiveL_Caudate..	nihss	kruskal	0.661328982504582			
191	5	0	Red-positiveL_Caudate..	nihss	kruskal	0.661328982504582			
192	6	0	Red-positiveL_Caudate..	nihss	kruskal	0.661328982504582			
193	1	0.5	Red-positiveL_Cerebellur	nihss	kruskal	0.0788773502826863			
194	2	3.33333333333333	Red-positiveL_Cerebellur	nihss	kruskal	0.0788773502826863			
195	3	0.875	Red-positiveL_Cerebellur	nihss	kruskal	0.0788773502826863			
196	4	0	Red-positiveL_Cerebellur	nihss	kruskal	0.0788773502826863			
197	5	1.5	Red-positiveL_Cerebellur	nihss	kruskal	0.0788773502826863			
198	6	3	Red-positiveL_Cerebellur	nihss	kruskal	0.0788773502826863			
199	1	0.75	Red-positiveL_Pallidum..	nihss	kruskal	0.509673307798573			
200	2	0.666666666666667	Red-positiveL_Pallidum..	nihss	kruskal	0.509673307798573			
201	3	0.625	Red-positiveL_Pallidum..	nihss	kruskal	0.509673307798573			
202	4	0	Red-positiveL_Pallidum..	nihss	kruskal	0.509673307798573			
203	5	0.5	Red-positiveL_Pallidum..	nihss	kruskal	0.509673307798573			
204	6	1	Red-positiveL_Pallidum..	nihss	kruskal	0.509673307798573			
205	1	1.75	Red-positiveL_Putamen..	nihss	kruskal	0.402270222815409			
206	2	0.333333333333333	Red-positiveL_Putamen..	nihss	kruskal	0.402270222815409			
207	3	0.75	Red-positiveL_Putamen..	nihss	kruskal	0.402270222815409			
208	4	1.25	Red-positiveL_Putamen..	nihss	kruskal	0.402270222815409			
209	5	1	Red-positiveL_Putamen..	nihss	kruskal	0.402270222815409			
210	6	0	Red-positiveL_Putamen..	nihss	kruskal	0.402270222815409			
211	1	0.5	Red-positiveL_Thalamus	nihss	kruskal	0.977527792551517			
212	2	0.666666666666667	Red-positiveL_Thalamus	nihss	kruskal	0.977527792551517			
213	3	1	Red-positiveL_Thalamus	nihss	kruskal	0.977527792551517			
214	4	0.75	Red-positiveL_Thalamus	nihss	kruskal	0.977527792551517			
215	5	0.5	Red-positiveL_Thalamus	nihss	kruskal	0.977527792551517			
216	6	1	Red-positiveL_Thalamus	nihss	kruskal	0.977527792551517			
217	1	1.5	Red-positiveL_VentralDC	nihss	kruskal	0.660087246482525			
218	2	2	Red-positiveL_VentralDC	nihss	kruskal	0.660087246482525			
219	3	2.125	Red-positiveL_VentralDC	nihss	kruskal	0.660087246482525			
220	4	0.5	Red-positiveL_VentralDC	nihss	kruskal	0.660087246482525			
221	5	0	Red-positiveL_VentralDC	nihss	kruskal	0.660087246482525			
222	6	1	Red-positiveL_VentralDC	nihss	kruskal	0.660087246482525			
223	1	0.5	Red-positiveR_Accumbe	nihss	kruskal	0.264859676621621			
224	2	0	Red-positiveR_Accumbe	nihss	kruskal	0.264859676621621			
225	3	0	Red-positiveR_Accumbe	nihss	kruskal	0.264859676621621			
226	4	0.5	Red-positiveR_Accumbe	nihss	kruskal	0.264859676621621			
227	5	0	Red-positiveR_Accumbe	nihss	kruskal	0.264859676621621			
228	6	0	Red-positiveR_Accumbe	nihss	kruskal	0.264859676621621			
229	1	0.25	Red-positiveR_Caudate..	nihss	kruskal	0.355536933716534			
230	2	1.33333333333333	Red-positiveR_Caudate..	nihss	kruskal	0.355536933716534			
231	3	1.125	Red-positiveR_Caudate..	nihss	kruskal	0.355536933716534			
232	4	1.75	Red-positiveR_Caudate..	nihss	kruskal	0.355536933716534			
233	5	0	Red-positiveR_Caudate..	nihss	kruskal	0.355536933716534			
234	6	0	Red-positiveR_Caudate..	nihss	kruskal	0.355536933716534			
235	1	0.25	Red-positiveR_Cerebellu	nihss	kruskal	0.389004198915358			
236	2	1.33333333333333	Red-positiveR_Cerebellu	nihss	kruskal	0.389004198915358			
237	3	1.125	Red-positiveR_Cerebellu	nihss	kruskal	0.389004198915358			
238	4	0.5	Red-positiveR_Cerebellu	nihss	kruskal	0.389004198915358			

239	5	3.5	Red-positiveR_Cerebellu	nihss	kruskal	0.389004198915358
240	6	7	Red-positiveR_Cerebellu	nihss	kruskal	0.389004198915358
241	1	1.25	Red-positiveR_Pallidum..	nihss	kruskal	0.604051210762831
242	2	0	Red-positiveR_Pallidum..	nihss	kruskal	0.604051210762831
243	3	0.5	Red-positiveR_Pallidum..	nihss	kruskal	0.604051210762831
244	4	1.25	Red-positiveR_Pallidum..	nihss	kruskal	0.604051210762831
245	5	2	Red-positiveR_Pallidum..	nihss	kruskal	0.604051210762831
246	6	1	Red-positiveR_Pallidum..	nihss	kruskal	0.604051210762831
247	1	2.25	Red-positiveR_Putamen.	nihss	kruskal	0.325663023123069
248	2	2	Red-positiveR_Putamen.	nihss	kruskal	0.325663023123069
249	3	0.625	Red-positiveR_Putamen.	nihss	kruskal	0.325663023123069
250	4	0	Red-positiveR_Putamen.	nihss	kruskal	0.325663023123069
251	5	1.5	Red-positiveR_Putamen.	nihss	kruskal	0.325663023123069
252	6	1	Red-positiveR_Putamen.	nihss	kruskal	0.325663023123069
253	1	2	Red-positiveR_Thalamus	nihss	kruskal	0.303529308793429
254	2	1.66666666666667	Red-positiveR_Thalamus	nihss	kruskal	0.303529308793429
255	3	0.125	Red-positiveR_Thalamus	nihss	kruskal	0.303529308793429
256	4	0.25	Red-positiveR_Thalamus	nihss	kruskal	0.303529308793429
257	5	0.5	Red-positiveR_Thalamus	nihss	kruskal	0.303529308793429
258	6	0	Red-positiveR_Thalamus	nihss	kruskal	0.303529308793429
259	1	0	Red-positiveR_VentralDC	nihss	kruskal	0.198857314823907
260	2	1	Red-positiveR_VentralDC	nihss	kruskal	0.198857314823907
261	3	0.375	Red-positiveR_VentralDC	nihss	kruskal	0.198857314823907
262	4	1.5	Red-positiveR_VentralDC	nihss	kruskal	0.198857314823907
263	5	0.5	Red-positiveR_VentralDC	nihss	kruskal	0.198857314823907
264	6	0	Red-positiveR_VentralDC	nihss	kruskal	0.198857314823907
265	1	8	Red-positiveL_cortical...4	nihss	kruskal	0.492279175295835
266	2	8	Red-positiveL_cortical...4	nihss	kruskal	0.492279175295835
267	3	12.5	Red-positiveL_cortical...4	nihss	kruskal	0.492279175295835
268	4	7	Red-positiveL_cortical...4	nihss	kruskal	0.492279175295835
269	5	15.5	Red-positiveL_cortical...4	nihss	kruskal	0.492279175295835
270	6	7	Red-positiveL_cortical...4	nihss	kruskal	0.492279175295835
271	1	6.75	Red-positiveR_cortical...	nihss	kruskal	0.884264761479565
272	2	8	Red-positiveR_cortical...	nihss	kruskal	0.884264761479565
273	3	9.75	Red-positiveR_cortical...	nihss	kruskal	0.884264761479565
274	4	7.75	Red-positiveR_cortical...	nihss	kruskal	0.884264761479565
275	5	9	Red-positiveR_cortical...	nihss	kruskal	0.884264761479565
276	6	4	Red-positiveR_cortical...	nihss	kruskal	0.884264761479565
277	1	6.25	Red-positiveL_subcortica	nihss	kruskal	0.824790738756724
278	2	8	Red-positiveL_subcortica	nihss	kruskal	0.824790738756724
279	3	6.875	Red-positiveL_subcortica	nihss	kruskal	0.824790738756724
280	4	4.5	Red-positiveL_subcortica	nihss	kruskal	0.824790738756724
281	5	3.5	Red-positiveL_subcortica	nihss	kruskal	0.824790738756724
282	6	6	Red-positiveL_subcortica	nihss	kruskal	0.824790738756724
283	1	6.5	Red-positiveR_subcortic	nihss	kruskal	0.821016503432645
284	2	7.33333333333333	Red-positiveR_subcortic	nihss	kruskal	0.821016503432645
285	3	3.875	Red-positiveR_subcortic	nihss	kruskal	0.821016503432645
286	4	5.75	Red-positiveR_subcortic	nihss	kruskal	0.821016503432645
287	5	8	Red-positiveR_subcortic	nihss	kruskal	0.821016503432645
288	6	9	Red-positiveR_subcortic	nihss	kruskal	0.821016503432645
289	1	1.5	Blue-negativeL_1...3	nihss	kruskal	0.625285773208374
290	2	2	Blue-negativeL_1...3	nihss	kruskal	0.625285773208374
291	3	1.25	Blue-negativeL_1...3	nihss	kruskal	0.625285773208374
292	4	0.5	Blue-negativeL_1...3	nihss	kruskal	0.625285773208374
293	5	0.5	Blue-negativeL_1...3	nihss	kruskal	0.625285773208374
294	6	0	Blue-negativeL_1...3	nihss	kruskal	0.625285773208374
295	1	1	Blue-negativeL_2...4	nihss	kruskal	0.38842984504525
296	2	0	Blue-negativeL_2...4	nihss	kruskal	0.38842984504525
297	3	0.375	Blue-negativeL_2...4	nihss	kruskal	0.38842984504525
298	4	0	Blue-negativeL_2...4	nihss	kruskal	0.38842984504525

299	5	0	Blue-negativeL_2...4	nihss	kruskal	0.38842984504525			
300	6	0	Blue-negativeL_2...4	nihss	kruskal	0.38842984504525			
301	1	0.25	Blue-negativeL_3a...5	nihss	kruskal	0.24131856488185			
302	2	0	Blue-negativeL_3a...5	nihss	kruskal	0.24131856488185			
303	3	0.125	Blue-negativeL_3a...5	nihss	kruskal	0.24131856488185			
304	4	0.5	Blue-negativeL_3a...5	nihss	kruskal	0.24131856488185			
305	5	0	Blue-negativeL_3a...5	nihss	kruskal	0.24131856488185			
306	6	1	Blue-negativeL_3a...5	nihss	kruskal	0.24131856488185			
307	1	0	Blue-negativeL_3b...6	nihss	kruskal	0.594294329644255			
308	2	0	Blue-negativeL_3b...6	nihss	kruskal	0.594294329644255			
309	3	0.125	Blue-negativeL_3b...6	nihss	kruskal	0.594294329644255			
310	4	0.75	Blue-negativeL_3b...6	nihss	kruskal	0.594294329644255			
311	5	0.5	Blue-negativeL_3b...6	nihss	kruskal	0.594294329644255			
312	6	0	Blue-negativeL_3b...6	nihss	kruskal	0.594294329644255			
313	1	0	Blue-negativeL_4...7	nihss	kruskal	0.796515802347148			
314	2	0	Blue-negativeL_4...7	nihss	kruskal	0.796515802347148			
315	3	0.125	Blue-negativeL_4...7	nihss	kruskal	0.796515802347148			
316	4	0.5	Blue-negativeL_4...7	nihss	kruskal	0.796515802347148			
317	5	0	Blue-negativeL_4...7	nihss	kruskal	0.796515802347148			
318	6	0	Blue-negativeL_4...7	nihss	kruskal	0.796515802347148			
319	1	0	Blue-negativeL_24dd...8	nihss	kruskal	0.34997523883583			
320	2	1.66666666666667	Blue-negativeL_24dd...8	nihss	kruskal	0.34997523883583			
321	3	0.625	Blue-negativeL_24dd...8	nihss	kruskal	0.34997523883583			
322	4	0.5	Blue-negativeL_24dd...8	nihss	kruskal	0.34997523883583			
323	5	0	Blue-negativeL_24dd...8	nihss	kruskal	0.34997523883583			
324	6	0	Blue-negativeL_24dd...8	nihss	kruskal	0.34997523883583			
325	1	1.25	Blue-negativeL_24dv...9	nihss	kruskal	0.207496233326414			
326	2	0.666666666666667	Blue-negativeL_24dv...9	nihss	kruskal	0.207496233326414			
327	3	0.125	Blue-negativeL_24dv...9	nihss	kruskal	0.207496233326414			
328	4	1	Blue-negativeL_24dv...9	nihss	kruskal	0.207496233326414			
329	5	0	Blue-negativeL_24dv...9	nihss	kruskal	0.207496233326414			
330	6	2	Blue-negativeL_24dv...9	nihss	kruskal	0.207496233326414			
331	1	0.5	Blue-negativeL_6a...10	nihss	kruskal	0.713473703582891			
332	2	0.333333333333333	Blue-negativeL_6a...10	nihss	kruskal	0.713473703582891			
333	3	0.125	Blue-negativeL_6a...10	nihss	kruskal	0.713473703582891			
334	4	0.5	Blue-negativeL_6a...10	nihss	kruskal	0.713473703582891			
335	5	0	Blue-negativeL_6a...10	nihss	kruskal	0.713473703582891			
336	6	0	Blue-negativeL_6a...10	nihss	kruskal	0.713473703582891			
337	1	0.25	Blue-negativeL_6d...11	nihss	kruskal	0.139884516407311			
338	2	1	Blue-negativeL_6d...11	nihss	kruskal	0.139884516407311			
339	3	0.75	Blue-negativeL_6d...11	nihss	kruskal	0.139884516407311			
340	4	0	Blue-negativeL_6d...11	nihss	kruskal	0.139884516407311			
341	5	0	Blue-negativeL_6d...11	nihss	kruskal	0.139884516407311			
342	6	1	Blue-negativeL_6d...11	nihss	kruskal	0.139884516407311			
343	1	0.25	Blue-negativeL_6ma...12	nihss	kruskal	0.629280009927692			
344	2	0	Blue-negativeL_6ma...12	nihss	kruskal	0.629280009927692			
345	3	0.625	Blue-negativeL_6ma...12	nihss	kruskal	0.629280009927692			
346	4	0.5	Blue-negativeL_6ma...12	nihss	kruskal	0.629280009927692			
347	5	0	Blue-negativeL_6ma...12	nihss	kruskal	0.629280009927692			
348	6	0	Blue-negativeL_6ma...12	nihss	kruskal	0.629280009927692			
349	1	0	Blue-negativeL_6mp...13	nihss	kruskal	NA			
350	2	0	Blue-negativeL_6mp...13	nihss	kruskal	NA			
351	3	0	Blue-negativeL_6mp...13	nihss	kruskal	NA			
352	4	0	Blue-negativeL_6mp...13	nihss	kruskal	NA			
353	5	0	Blue-negativeL_6mp...13	nihss	kruskal	NA			
354	6	0	Blue-negativeL_6mp...13	nihss	kruskal	NA			
355	1	0.5	Blue-negativeL_SCEF...14	nihss	kruskal	0.420986407920185			
356	2	0	Blue-negativeL_SCEF...14	nihss	kruskal	0.420986407920185			
357	3	0.25	Blue-negativeL_SCEF...14	nihss	kruskal	0.420986407920185			
358	4	0	Blue-negativeL_SCEF...14	nihss	kruskal	0.420986407920185			

359	5	0	Blue-negativeL_SCEF...14	nihss	kruskal	0.420986407920185			
360	6	0	Blue-negativeL_SCEF...14	nihss	kruskal	0.420986407920185			
361	1	2	Blue-negativeL_SFL...15	nihss	kruskal	0.761610568925241			
362	2	0.333333333333333	Blue-negativeL_SFL...15	nihss	kruskal	0.761610568925241			
363	3	0.875	Blue-negativeL_SFL...15	nihss	kruskal	0.761610568925241			
364	4	0.75	Blue-negativeL_SFL...15	nihss	kruskal	0.761610568925241			
365	5	0	Blue-negativeL_SFL...15	nihss	kruskal	0.761610568925241			
366	6	0	Blue-negativeL_SFL...15	nihss	kruskal	0.761610568925241			
367	1	0.5	Blue-negativeL_6r...16	nihss	kruskal	0.191773169475663			
368	2	0	Blue-negativeL_6r...16	nihss	kruskal	0.191773169475663			
369	3	0.25	Blue-negativeL_6r...16	nihss	kruskal	0.191773169475663			
370	4	0	Blue-negativeL_6r...16	nihss	kruskal	0.191773169475663			
371	5	0	Blue-negativeL_6r...16	nihss	kruskal	0.191773169475663			
372	6	1	Blue-negativeL_6r...16	nihss	kruskal	0.191773169475663			
373	1	0.25	Blue-negativeL_6v...17	nihss	kruskal	0.549844530743605			
374	2	0.333333333333333	Blue-negativeL_6v...17	nihss	kruskal	0.549844530743605			
375	3	0.625	Blue-negativeL_6v...17	nihss	kruskal	0.549844530743605			
376	4	0.75	Blue-negativeL_6v...17	nihss	kruskal	0.549844530743605			
377	5	0	Blue-negativeL_6v...17	nihss	kruskal	0.549844530743605			
378	6	0	Blue-negativeL_6v...17	nihss	kruskal	0.549844530743605			
379	1	1.75	Blue-negativeR_1...18	nihss	kruskal	0.470882385522027			
380	2	0.333333333333333	Blue-negativeR_1...18	nihss	kruskal	0.470882385522027			
381	3	1.25	Blue-negativeR_1...18	nihss	kruskal	0.470882385522027			
382	4	1	Blue-negativeR_1...18	nihss	kruskal	0.470882385522027			
383	5	0.5	Blue-negativeR_1...18	nihss	kruskal	0.470882385522027			
384	6	1	Blue-negativeR_1...18	nihss	kruskal	0.470882385522027			
385	1	1	Blue-negativeR_2...19	nihss	kruskal	0.556303489407597			
386	2	0	Blue-negativeR_2...19	nihss	kruskal	0.556303489407597			
387	3	0.375	Blue-negativeR_2...19	nihss	kruskal	0.556303489407597			
388	4	0	Blue-negativeR_2...19	nihss	kruskal	0.556303489407597			
389	5	0	Blue-negativeR_2...19	nihss	kruskal	0.556303489407597			
390	6	0	Blue-negativeR_2...19	nihss	kruskal	0.556303489407597			
391	1	0.25	Blue-negativeR_3a...20	nihss	kruskal	0.455671246331592			
392	2	0.333333333333333	Blue-negativeR_3a...20	nihss	kruskal	0.455671246331592			
393	3	0.625	Blue-negativeR_3a...20	nihss	kruskal	0.455671246331592			
394	4	0	Blue-negativeR_3a...20	nihss	kruskal	0.455671246331592			
395	5	0	Blue-negativeR_3a...20	nihss	kruskal	0.455671246331592			
396	6	0	Blue-negativeR_3a...20	nihss	kruskal	0.455671246331592			
397	1	0.5	Blue-negativeR_3b...21	nihss	kruskal	0.68589361146684			
398	2	0.333333333333333	Blue-negativeR_3b...21	nihss	kruskal	0.68589361146684			
399	3	0.875	Blue-negativeR_3b...21	nihss	kruskal	0.68589361146684			
400	4	0	Blue-negativeR_3b...21	nihss	kruskal	0.68589361146684			
401	5	0	Blue-negativeR_3b...21	nihss	kruskal	0.68589361146684			
402	6	0	Blue-negativeR_3b...21	nihss	kruskal	0.68589361146684			
403	1	0.75	Blue-negativeR_4...22	nihss	kruskal	0.179021444984202			
404	2	1.33333333333333	Blue-negativeR_4...22	nihss	kruskal	0.179021444984202			
405	3	0.125	Blue-negativeR_4...22	nihss	kruskal	0.179021444984202			
406	4	0	Blue-negativeR_4...22	nihss	kruskal	0.179021444984202			
407	5	0	Blue-negativeR_4...22	nihss	kruskal	0.179021444984202			
408	6	0	Blue-negativeR_4...22	nihss	kruskal	0.179021444984202			
409	1	1.25	Blue-negativeR_24dd...23	nihss	kruskal	0.501447060543386			
410	2	0.666666666666667	Blue-negativeR_24dd...23	nihss	kruskal	0.501447060543386			
411	3	0.875	Blue-negativeR_24dd...23	nihss	kruskal	0.501447060543386			
412	4	0.75	Blue-negativeR_24dd...23	nihss	kruskal	0.501447060543386			
413	5	0	Blue-negativeR_24dd...23	nihss	kruskal	0.501447060543386			
414	6	0	Blue-negativeR_24dd...23	nihss	kruskal	0.501447060543386			
415	1	0	Blue-negativeR_24dv...24	nihss	kruskal	0.332118874887682			
416	2	0.333333333333333	Blue-negativeR_24dv...24	nihss	kruskal	0.332118874887682			
417	3	0.625	Blue-negativeR_24dv...24	nihss	kruskal	0.332118874887682			
418	4	0.5	Blue-negativeR_24dv...24	nihss	kruskal	0.332118874887682			

419	5	0	Blue-negativeR_24dv...24	nihss	kruskal	0.332118874887682			
420	6	0	Blue-negativeR_24dv...24	nihss	kruskal	0.332118874887682			
421	1	0.5	Blue-negativeR_6a...25	nihss	kruskal	0.665102303494867			
422	2	0.333333333333333	Blue-negativeR_6a...25	nihss	kruskal	0.665102303494867			
423	3	0.375	Blue-negativeR_6a...25	nihss	kruskal	0.665102303494867			
424	4	0.25	Blue-negativeR_6a...25	nihss	kruskal	0.665102303494867			
425	5	0	Blue-negativeR_6a...25	nihss	kruskal	0.665102303494867			
426	6	1	Blue-negativeR_6a...25	nihss	kruskal	0.665102303494867			
427	1	0	Blue-negativeR_6d...26	nihss	kruskal	0.451606274537042			
428	2	0.333333333333333	Blue-negativeR_6d...26	nihss	kruskal	0.451606274537042			
429	3	0.5	Blue-negativeR_6d...26	nihss	kruskal	0.451606274537042			
430	4	0.25	Blue-negativeR_6d...26	nihss	kruskal	0.451606274537042			
431	5	0	Blue-negativeR_6d...26	nihss	kruskal	0.451606274537042			
432	6	1	Blue-negativeR_6d...26	nihss	kruskal	0.451606274537042			
433	1	1.5	Blue-negativeR_6ma...27	nihss	kruskal	0.468798582556077			
434	2	1.33333333333333	Blue-negativeR_6ma...27	nihss	kruskal	0.468798582556077			
435	3	0.375	Blue-negativeR_6ma...27	nihss	kruskal	0.468798582556077			
436	4	1	Blue-negativeR_6ma...27	nihss	kruskal	0.468798582556077			
437	5	0	Blue-negativeR_6ma...27	nihss	kruskal	0.468798582556077			
438	6	2	Blue-negativeR_6ma...27	nihss	kruskal	0.468798582556077			
439	1	0	Blue-negativeR_6mp...28	nihss	kruskal	0.275121683221223			
440	2	0.333333333333333	Blue-negativeR_6mp...28	nihss	kruskal	0.275121683221223			
441	3	0	Blue-negativeR_6mp...28	nihss	kruskal	0.275121683221223			
442	4	0	Blue-negativeR_6mp...28	nihss	kruskal	0.275121683221223			
443	5	0	Blue-negativeR_6mp...28	nihss	kruskal	0.275121683221223			
444	6	0	Blue-negativeR_6mp...28	nihss	kruskal	0.275121683221223			
445	1	0.5	Blue-negativeR_SCEF...2	nihss	kruskal	0.479883438113305			
446	2	0	Blue-negativeR_SCEF...2	nihss	kruskal	0.479883438113305			
447	3	0	Blue-negativeR_SCEF...2	nihss	kruskal	0.479883438113305			
448	4	0	Blue-negativeR_SCEF...2	nihss	kruskal	0.479883438113305			
449	5	0	Blue-negativeR_SCEF...2	nihss	kruskal	0.479883438113305			
450	6	0	Blue-negativeR_SCEF...2	nihss	kruskal	0.479883438113305			
451	1	0.75	Blue-negativeR_SFL...30	nihss	kruskal	0.459039475544525			
452	2	0	Blue-negativeR_SFL...30	nihss	kruskal	0.459039475544525			
453	3	0.75	Blue-negativeR_SFL...30	nihss	kruskal	0.459039475544525			
454	4	0.25	Blue-negativeR_SFL...30	nihss	kruskal	0.459039475544525			
455	5	0	Blue-negativeR_SFL...30	nihss	kruskal	0.459039475544525			
456	6	0	Blue-negativeR_SFL...30	nihss	kruskal	0.459039475544525			
457	1	1.25	Blue-negativeR_6r...31	nihss	kruskal	0.250302067742114			
458	2	0.666666666666667	Blue-negativeR_6r...31	nihss	kruskal	0.250302067742114			
459	3	0.625	Blue-negativeR_6r...31	nihss	kruskal	0.250302067742114			
460	4	0	Blue-negativeR_6r...31	nihss	kruskal	0.250302067742114			
461	5	0	Blue-negativeR_6r...31	nihss	kruskal	0.250302067742114			
462	6	0	Blue-negativeR_6r...31	nihss	kruskal	0.250302067742114			
463	1	1	Blue-negativeR_6v...32	nihss	kruskal	0.231219605273691			
464	2	1	Blue-negativeR_6v...32	nihss	kruskal	0.231219605273691			
465	3	0.5	Blue-negativeR_6v...32	nihss	kruskal	0.231219605273691			
466	4	0	Blue-negativeR_6v...32	nihss	kruskal	0.231219605273691			
467	5	0	Blue-negativeR_6v...32	nihss	kruskal	0.231219605273691			
468	6	2	Blue-negativeR_6v...32	nihss	kruskal	0.231219605273691			
469	1	0	Blue-negativeL_Accumbe	nihss	kruskal	0.70319788722226			
470	2	0.333333333333333	Blue-negativeL_Accumbe	nihss	kruskal	0.70319788722226			
471	3	0.25	Blue-negativeL_Accumbe	nihss	kruskal	0.70319788722226			
472	4	0	Blue-negativeL_Accumbe	nihss	kruskal	0.70319788722226			
473	5	0	Blue-negativeL_Accumbe	nihss	kruskal	0.70319788722226			
474	6	0	Blue-negativeL_Accumbe	nihss	kruskal	0.70319788722226			
475	1	1	Blue-negativeL_Caudate.	nihss	kruskal	0.361526016466503			
476	2	0.333333333333333	Blue-negativeL_Caudate.	nihss	kruskal	0.361526016466503			
477	3	0.75	Blue-negativeL_Caudate.	nihss	kruskal	0.361526016466503			
478	4	0	Blue-negativeL_Caudate.	nihss	kruskal	0.361526016466503			

479	5	0.5	Blue-negativeL_Caudate.	nihss	kruskal	0.361526016466503			
480	6	0	Blue-negativeL_Caudate.	nihss	kruskal	0.361526016466503			
481	1	1.75	Blue-negativeL_Cerebellu	nihss	kruskal	0.34962464384309			
482	2	2	Blue-negativeL_Cerebellu	nihss	kruskal	0.34962464384309			
483	3	2.125	Blue-negativeL_Cerebellu	nihss	kruskal	0.34962464384309			
484	4	0.25	Blue-negativeL_Cerebellu	nihss	kruskal	0.34962464384309			
485	5	0	Blue-negativeL_Cerebellu	nihss	kruskal	0.34962464384309			
486	6	3	Blue-negativeL_Cerebellu	nihss	kruskal	0.34962464384309			
487	1	1.25	Blue-negativeL_Pallidum	nihss	kruskal	0.265503109850938			
488	2	0.333333333333333	Blue-negativeL_Pallidum	nihss	kruskal	0.265503109850938			
489	3	1.5	Blue-negativeL_Pallidum	nihss	kruskal	0.265503109850938			
490	4	0.5	Blue-negativeL_Pallidum	nihss	kruskal	0.265503109850938			
491	5	0	Blue-negativeL_Pallidum	nihss	kruskal	0.265503109850938			
492	6	0	Blue-negativeL_Pallidum	nihss	kruskal	0.265503109850938			
493	1	0.5	Blue-negativeL_Putamen	nihss	kruskal	0.685665031294243			
494	2	1.33333333333333	Blue-negativeL_Putamen	nihss	kruskal	0.685665031294243			
495	3	0.5	Blue-negativeL_Putamen	nihss	kruskal	0.685665031294243			
496	4	0.75	Blue-negativeL_Putamen	nihss	kruskal	0.685665031294243			
497	5	0	Blue-negativeL_Putamen	nihss	kruskal	0.685665031294243			
498	6	0	Blue-negativeL_Putamen	nihss	kruskal	0.685665031294243			
499	1	1.75	Blue-negativeL_Thalamus	nihss	kruskal	0.279292427143623			
500	2	0.333333333333333	Blue-negativeL_Thalamus	nihss	kruskal	0.279292427143623			
501	3	1.375	Blue-negativeL_Thalamus	nihss	kruskal	0.279292427143623			
502	4	0	Blue-negativeL_Thalamus	nihss	kruskal	0.279292427143623			
503	5	0	Blue-negativeL_Thalamus	nihss	kruskal	0.279292427143623			
504	6	0	Blue-negativeL_Thalamus	nihss	kruskal	0.279292427143623			
505	1	1	Blue-negativeL_VentralD(	nihss	kruskal	0.356616857901304			
506	2	1.66666666666667	Blue-negativeL_VentralD(	nihss	kruskal	0.356616857901304			
507	3	0.75	Blue-negativeL_VentralD(	nihss	kruskal	0.356616857901304			
508	4	0	Blue-negativeL_VentralD(	nihss	kruskal	0.356616857901304			
509	5	0	Blue-negativeL_VentralD(	nihss	kruskal	0.356616857901304			
510	6	0	Blue-negativeL_VentralD(	nihss	kruskal	0.356616857901304			
511	1	0.25	Blue-negativeR_Accumb	nihss	kruskal	0.915064544742432			
512	2	0.333333333333333	Blue-negativeR_Accumb	nihss	kruskal	0.915064544742432			
513	3	0.125	Blue-negativeR_Accumb	nihss	kruskal	0.915064544742432			
514	4	0.25	Blue-negativeR_Accumb	nihss	kruskal	0.915064544742432			
515	5	0	Blue-negativeR_Accumb	nihss	kruskal	0.915064544742432			
516	6	0	Blue-negativeR_Accumb	nihss	kruskal	0.915064544742432			
517	1	0.5	Blue-negativeR_Caudate	nihss	kruskal	0.791373494894993			
518	2	0.333333333333333	Blue-negativeR_Caudate	nihss	kruskal	0.791373494894993			
519	3	0.75	Blue-negativeR_Caudate	nihss	kruskal	0.791373494894993			
520	4	1.25	Blue-negativeR_Caudate	nihss	kruskal	0.791373494894993			
521	5	0	Blue-negativeR_Caudate	nihss	kruskal	0.791373494894993			
522	6	0	Blue-negativeR_Caudate	nihss	kruskal	0.791373494894993			
523	1	1.75	Blue-negativeR_Cerebell	nihss	kruskal	0.692131087782116			
524	2	1.33333333333333	Blue-negativeR_Cerebell	nihss	kruskal	0.692131087782116			
525	3	1.375	Blue-negativeR_Cerebell	nihss	kruskal	0.692131087782116			
526	4	0.25	Blue-negativeR_Cerebell	nihss	kruskal	0.692131087782116			
527	5	0.5	Blue-negativeR_Cerebell	nihss	kruskal	0.692131087782116			
528	6	0	Blue-negativeR_Cerebell	nihss	kruskal	0.692131087782116			
529	1	0.5	Blue-negativeR_Pallidum	nihss	kruskal	0.741813597114429			
530	2	0.333333333333333	Blue-negativeR_Pallidum	nihss	kruskal	0.741813597114429			
531	3	0.375	Blue-negativeR_Pallidum	nihss	kruskal	0.741813597114429			
532	4	0.5	Blue-negativeR_Pallidum	nihss	kruskal	0.741813597114429			
533	5	0	Blue-negativeR_Pallidum	nihss	kruskal	0.741813597114429			
534	6	1	Blue-negativeR_Pallidum	nihss	kruskal	0.741813597114429			
535	1	0.5	Blue-negativeR_Putamer	nihss	kruskal	0.889198340536124			
536	2	0.666666666666667	Blue-negativeR_Putamer	nihss	kruskal	0.889198340536124			
537	3	0.75	Blue-negativeR_Putamer	nihss	kruskal	0.889198340536124			
538	4	0.25	Blue-negativeR_Putamer	nihss	kruskal	0.889198340536124			

539	5	0	Blue-negativeR_Putamer	nihss	kruskal	0.889198340536124			
540	6	0	Blue-negativeR_Putamer	nihss	kruskal	0.889198340536124			
541	1	0.5	Blue-negativeR_Thalamu	nihss	kruskal	0.769472171535226			
542	2	0.666666666666667	Blue-negativeR_Thalamu	nihss	kruskal	0.769472171535226			
543	3	0.5	Blue-negativeR_Thalamu	nihss	kruskal	0.769472171535226			
544	4	0.25	Blue-negativeR_Thalamu	nihss	kruskal	0.769472171535226			
545	5	0	Blue-negativeR_Thalamu	nihss	kruskal	0.769472171535226			
546	6	0	Blue-negativeR_Thalamu	nihss	kruskal	0.769472171535226			
547	1	0	Blue-negativeR_VentralD	nihss	kruskal	0.630017654321076			
548	2	1	Blue-negativeR_VentralD	nihss	kruskal	0.630017654321076			
549	3	0.75	Blue-negativeR_VentralD	nihss	kruskal	0.630017654321076			
550	4	0.5	Blue-negativeR_VentralD	nihss	kruskal	0.630017654321076			
551	5	0.5	Blue-negativeR_VentralD	nihss	kruskal	0.630017654321076			
552	6	0	Blue-negativeR_VentralD	nihss	kruskal	0.630017654321076			
553	1	8.25	Blue-negativeL_cortical...	nihss	kruskal	0.368613973433101			
554	2	6	Blue-negativeL_cortical...	nihss	kruskal	0.368613973433101			
555	3	6.25	Blue-negativeL_cortical...	nihss	kruskal	0.368613973433101			
556	4	4	Blue-negativeL_cortical...	nihss	kruskal	0.368613973433101			
557	5	1	Blue-negativeL_cortical...	nihss	kruskal	0.368613973433101			
558	6	5	Blue-negativeL_cortical...	nihss	kruskal	0.368613973433101			
559	1	11	Blue-negativeR_cortical...	nihss	kruskal	0.335105175904348			
560	2	7.33333333333333	Blue-negativeR_cortical...	nihss	kruskal	0.335105175904348			
561	3	7.875	Blue-negativeR_cortical...	nihss	kruskal	0.335105175904348			
562	4	4	Blue-negativeR_cortical...	nihss	kruskal	0.335105175904348			
563	5	0.5	Blue-negativeR_cortical...	nihss	kruskal	0.335105175904348			
564	6	7	Blue-negativeR_cortical...	nihss	kruskal	0.335105175904348			
565	1	6.25	Blue-negativeL_subcortic	nihss	kruskal	0.408274358077545			
566	2	6	Blue-negativeL_subcortic	nihss	kruskal	0.408274358077545			
567	3	7	Blue-negativeL_subcortic	nihss	kruskal	0.408274358077545			
568	4	2.75	Blue-negativeL_subcortic	nihss	kruskal	0.408274358077545			
569	5	0.5	Blue-negativeL_subcortic	nihss	kruskal	0.408274358077545			
570	6	3	Blue-negativeL_subcortic	nihss	kruskal	0.408274358077545			
571	1	4	Blue-negativeR_subcortic	nihss	kruskal	0.614526897333525			
572	2	4.66666666666667	Blue-negativeR_subcortic	nihss	kruskal	0.614526897333525			
573	3	4.625	Blue-negativeR_subcortic	nihss	kruskal	0.614526897333525			
574	4	3.25	Blue-negativeR_subcortic	nihss	kruskal	0.614526897333525			
575	5	1	Blue-negativeR_subcortic	nihss	kruskal	0.614526897333525			
576	6	1	Blue-negativeR_subcortic	nihss	kruskal	0.614526897333525			
577	1	1.88888888888889	Red-positiveL_1...3	fm	wilcox	0.430412353115454			
578	2	2.25	Red-positiveL_1...3	fm	wilcox	0.430412353115454			
579	1	0.5	Red-positiveL_2...4	fm	wilcox	0.584168767522232			
580	2	0.75	Red-positiveL_2...4	fm	wilcox	0.584168767522232			
581	1	0.722222222222222	Red-positiveL_3a...5	fm	wilcox	1			
582	2	0.5	Red-positiveL_3a...5	fm	wilcox	1			
583	1	0.5	Red-positiveL_3b...6	fm	wilcox	0.117750414605118			
584	2	0	Red-positiveL_3b...6	fm	wilcox	0.117750414605118			
585	1	0.222222222222222	Red-positiveL_4...7	fm	wilcox	0.339727775866098			
586	2	0	Red-positiveL_4...7	fm	wilcox	0.339727775866098			
587	1	0.944444444444444	Red-positiveL_24dd...8	fm	wilcox	0.30239907081377			
588	2	0.25	Red-positiveL_24dd...8	fm	wilcox	0.30239907081377			
589	1	1.38888888888889	Red-positiveL_24dv...9	fm	wilcox	1			
590	2	1	Red-positiveL_24dv...9	fm	wilcox	1			
591	1	0.333333333333333	Red-positiveL_6a...10	fm	wilcox	1			
592	2	0.25	Red-positiveL_6a...10	fm	wilcox	1			
593	1	1	Red-positiveL_6d...11	fm	wilcox	0.23089120140959			
594	2	0.25	Red-positiveL_6d...11	fm	wilcox	0.23089120140959			
595	1	0.888888888888889	Red-positiveL_6ma...12	fm	wilcox	0.387672098787085			
596	2	0.25	Red-positiveL_6ma...12	fm	wilcox	0.387672098787085			
597	1	0.277777777777778	Red-positiveL_6mp...13	fm	wilcox	0.341549864797697			
598	2	0	Red-positiveL_6mp...13	fm	wilcox	0.341549864797697			

599	1	0.277777777777778	Red-positiveL_SCEF...14	fm	wilcox	1			
600	2	0.25	Red-positiveL_SCEF...14	fm	wilcox	1			
601	1	0.444444444444444	Red-positiveL_SFL...15	fm	wilcox	0.342154042087154			
602	2	0	Red-positiveL_SFL...15	fm	wilcox	0.342154042087154			
603	1	0.277777777777778	Red-positiveL_6r...16	fm	wilcox	0.0921769922980364			
604	2	0.75	Red-positiveL_6r...16	fm	wilcox	0.0921769922980364			
605	1	1.33333333333333	Red-positiveL_6v...17	fm	wilcox	0.624790430951147			
606	2	1.5	Red-positiveL_6v...17	fm	wilcox	0.624790430951147			
607	1	1.66666666666667	Red-positiveR_1...18	fm	wilcox	0.171244503197586			
608	2	0.5	Red-positiveR_1...18	fm	wilcox	0.171244503197586			
609	1	0.388888888888889	Red-positiveR_2...19	fm	wilcox	0.342455537036634			
610	2	0	Red-positiveR_2...19	fm	wilcox	0.342455537036634			
611	1	0.611111111111111	Red-positiveR_3a...20	fm	wilcox	0.827964717617683			
612	2	0.25	Red-positiveR_3a...20	fm	wilcox	0.827964717617683			
613	1	0.5	Red-positiveR_3b...21	fm	wilcox	0.413825113502138			
614	2	0.5	Red-positiveR_3b...21	fm	wilcox	0.413825113502138			
615	1	0.0555555555555556	Red-positiveR_4...22	fm	wilcox	0.232430481974116			
616	2	0.75	Red-positiveR_4...22	fm	wilcox	0.232430481974116			
617	1	0.722222222222222	Red-positiveR_24dd...23	fm	wilcox	0.211884737518174			
618	2	0	Red-positiveR_24dd...23	fm	wilcox	0.211884737518174			
619	1	0.444444444444444	Red-positiveR_24dv...24	fm	wilcox	0.64972262878551			
620	2	0.5	Red-positiveR_24dv...24	fm	wilcox	0.64972262878551			
621	1	0.222222222222222	Red-positiveR_6a...25	fm	wilcox	0.339727775866098			
622	2	0	Red-positiveR_6a...25	fm	wilcox	0.339727775866098			
623	1	0.722222222222222	Red-positiveR_6d...26	fm	wilcox	0.438930752990269			
624	2	0.25	Red-positiveR_6d...26	fm	wilcox	0.438930752990269			
625	1	0.777777777777778	Red-positiveR_6ma...27	fm	wilcox	0.118032452997732			
626	2	1.25	Red-positiveR_6ma...27	fm	wilcox	0.118032452997732			
627	1	0.111111111111111	Red-positiveR_6mp...28	fm	wilcox	0.550014235442413			
628	2	0	Red-positiveR_6mp...28	fm	wilcox	0.550014235442413			
629	1	0.5	Red-positiveR_SCEF...29	fm	wilcox	0.581828593963698			
630	2	0.25	Red-positiveR_SCEF...29	fm	wilcox	0.581828593963698			
631	1	0.611111111111111	Red-positiveR_SFL...30	fm	wilcox	0.913236247319931			
632	2	1.75	Red-positiveR_SFL...30	fm	wilcox	0.913236247319931			
633	1	0.166666666666667	Red-positiveR_6r...31	fm	wilcox	0.431362565270578			
634	2	0	Red-positiveR_6r...31	fm	wilcox	0.431362565270578			
635	1	1.11111111111111	Red-positiveR_6v...32	fm	wilcox	0.492577822849741			
636	2	0.75	Red-positiveR_6v...32	fm	wilcox	0.492577822849741			
637	1	0.333333333333333	Red-positiveL_Accumber	fm	wilcox	0.861669638217797			
638	2	0.5	Red-positiveL_Accumber	fm	wilcox	0.861669638217797			
639	1	0.777777777777778	Red-positiveL_Caudate..	fm	wilcox	0.447525539866669			
640	2	1	Red-positiveL_Caudate..	fm	wilcox	0.447525539866669			
641	1	1.16666666666667	Red-positiveL_Cerebellur	fm	wilcox	0.963439828399055			
642	2	1	Red-positiveL_Cerebellur	fm	wilcox	0.963439828399055			
643	1	0.5	Red-positiveL_Pallidum..	fm	wilcox	0.666583114091637			
644	2	0.75	Red-positiveL_Pallidum..	fm	wilcox	0.666583114091637			
645	1	1.05555555555556	Red-positiveL_Putamen..	fm	wilcox	0.382866321123493			
646	2	0.5	Red-positiveL_Putamen..	fm	wilcox	0.382866321123493			
647	1	0.722222222222222	Red-positiveL_Thalamus.	fm	wilcox	0.460214035803811			
648	2	1	Red-positiveL_Thalamus.	fm	wilcox	0.460214035803811			
649	1	1.72222222222222	Red-positiveL_VentralDC	fm	wilcox	0.244587771562752			
650	2	0.25	Red-positiveL_VentralDC	fm	wilcox	0.244587771562752			
651	1	0.166666666666667	Red-positiveR_Accumbe	fm	wilcox	0.568067955844146			
652	2	0.25	Red-positiveR_Accumbe	fm	wilcox	0.568067955844146			
653	1	0.944444444444444	Red-positiveR_Caudate..	fm	wilcox	0.925557733182569			
654	2	1	Red-positiveR_Caudate..	fm	wilcox	0.925557733182569			
655	1	1.27777777777778	Red-positiveR_Cerebellu	fm	wilcox	0.774016146687544			
656	2	1.75	Red-positiveR_Cerebellu	fm	wilcox	0.774016146687544			
657	1	0.944444444444444	Red-positiveR_Pallidum..	fm	wilcox	0.718064071857831			
658	2	0.5	Red-positiveR_Pallidum..	fm	wilcox	0.718064071857831			

659	1	1.22222222222222	Red-positiveR_Putamen.	fm	wilcox	0.887739653840375			
660	2	0.5	Red-positiveR_Putamen.	fm	wilcox	0.887739653840375			
661	1	0.722222222222222	Red-positiveR_Thalamus	fm	wilcox	0.876776911518985			
662	2	0.75	Red-positiveR_Thalamus	fm	wilcox	0.876776911518985			
663	1	0.5	Red-positiveR_VentralDC	fm	wilcox	0.561577454035133			
664	2	1	Red-positiveR_VentralDC	fm	wilcox	0.561577454035133			
665	1	10.8333333333333	Red-positiveL_cortical...4	fm	wilcox	0.282212113829251			
666	2	6.75	Red-positiveL_cortical...4	fm	wilcox	0.282212113829251			
667	1	8.61111111111111	Red-positiveR_cortical...4	fm	wilcox	0.549446547058541			
668	2	6.75	Red-positiveR_cortical...4	fm	wilcox	0.549446547058541			
669	1	6.22222222222222	Red-positiveL_subcortica	fm	wilcox	0.965905422277843			
670	2	5.75	Red-positiveL_subcortica	fm	wilcox	0.965905422277843			
671	1	5.77777777777778	Red-positiveR_subcortica	fm	wilcox	0.5488767183829			
672	2	5.75	Red-positiveR_subcortica	fm	wilcox	0.5488767183829			
673	1	1.11111111111111	Blue-negativeL_1...3	fm	wilcox	0.89330037488895			
674	2	1.25	Blue-negativeL_1...3	fm	wilcox	0.89330037488895			
675	1	0.388888888888889	Blue-negativeL_2...4	fm	wilcox	0.341549864797697			
676	2	0	Blue-negativeL_2...4	fm	wilcox	0.341549864797697			
677	1	0.166666666666667	Blue-negativeL_3a...5	fm	wilcox	0.177886166388196			
678	2	0.5	Blue-negativeL_3a...5	fm	wilcox	0.177886166388196			
679	1	0.277777777777778	Blue-negativeL_3b...6	fm	wilcox	0.432461521741892			
680	2	0	Blue-negativeL_3b...6	fm	wilcox	0.432461521741892			
681	1	0.166666666666667	Blue-negativeL_4...7	fm	wilcox	0.550466854058989			
682	2	0	Blue-negativeL_4...7	fm	wilcox	0.550466854058989			
683	1	0.666666666666667	Blue-negativeL_24dd...8	fm	wilcox	0.21167486517783			
684	2	0	Blue-negativeL_24dd...8	fm	wilcox	0.21167486517783			
685	1	0.5	Blue-negativeL_24dv...9	fm	wilcox	0.302276516934014			
686	2	1.25	Blue-negativeL_24dv...9	fm	wilcox	0.302276516934014			
687	1	0.166666666666667	Blue-negativeL_6a...10	fm	wilcox	0.129786050945976			
688	2	0.75	Blue-negativeL_6a...10	fm	wilcox	0.129786050945976			
689	1	0.5	Blue-negativeL_6d...11	fm	wilcox	0.726099595557261			
690	2	0.5	Blue-negativeL_6d...11	fm	wilcox	0.726099595557261			
691	1	0.388888888888889	Blue-negativeL_6ma...12	fm	wilcox	0.870115477417024			
692	2	0.25	Blue-negativeL_6ma...12	fm	wilcox	0.870115477417024			
693	1	0	Blue-negativeL_6mp...13	fm	wilcox	NA			
694	2	0	Blue-negativeL_6mp...13	fm	wilcox	NA			
695	1	0.166666666666667	Blue-negativeL_SCEF...1	fm	wilcox	0.750307122981635			
696	2	0.25	Blue-negativeL_SCEF...1	fm	wilcox	0.750307122981635			
697	1	0.888888888888889	Blue-negativeL_SFL...15	fm	wilcox	0.836144270798708			
698	2	0.75	Blue-negativeL_SFL...15	fm	wilcox	0.836144270798708			
699	1	0.222222222222222	Blue-negativeL_6r...16	fm	wilcox	0.953288298869363			
700	2	0.25	Blue-negativeL_6r...16	fm	wilcox	0.953288298869363			
701	1	0.5	Blue-negativeL_6v...17	fm	wilcox	0.490555029031673			
702	2	0.25	Blue-negativeL_6v...17	fm	wilcox	0.490555029031673			
703	1	1.05555555555556	Blue-negativeR_1...18	fm	wilcox	0.554347415622159			
704	2	1.25	Blue-negativeR_1...18	fm	wilcox	0.554347415622159			
705	1	0.166666666666667	Blue-negativeR_2...19	fm	wilcox	0.611974313539991			
706	2	1	Blue-negativeR_2...19	fm	wilcox	0.611974313539991			
707	1	0.388888888888889	Blue-negativeR_3a...20	fm	wilcox	0.208085712297209			
708	2	0	Blue-negativeR_3a...20	fm	wilcox	0.208085712297209			
709	1	0.555555555555556	Blue-negativeR_3b...21	fm	wilcox	0.270279331455849			
710	2	0	Blue-negativeR_3b...21	fm	wilcox	0.270279331455849			
711	1	0.388888888888889	Blue-negativeR_4...22	fm	wilcox	1			
712	2	0.25	Blue-negativeR_4...22	fm	wilcox	1			
713	1	0.888888888888889	Blue-negativeR_24dd...2	fm	wilcox	0.261054189579657			
714	2	0.25	Blue-negativeR_24dd...2	fm	wilcox	0.261054189579657			
715	1	0.333333333333333	Blue-negativeR_24dv...2	fm	wilcox	1			
716	2	0.5	Blue-negativeR_24dv...2	fm	wilcox	1			
717	1	0.333333333333333	Blue-negativeR_6a...25	fm	wilcox	0.497128293502778			
718	2	0.5	Blue-negativeR_6a...25	fm	wilcox	0.497128293502778			

719	1	0.333333333333333	Blue-negativeR_6d...26	fm	wilcox	1			
720	2	0.25	Blue-negativeR_6d...26	fm	wilcox	1			
721	1	0.666666666666667	Blue-negativeR_6ma...27	fm	wilcox	0.146582811732832			
722	2	1.75	Blue-negativeR_6ma...27	fm	wilcox	0.146582811732832			
723	1	0.0555555555555556	Blue-negativeR_6mp...28	fm	wilcox	0.723673609831763			
724	2	0	Blue-negativeR_6mp...28	fm	wilcox	0.723673609831763			
725	1	0.111111111111111	Blue-negativeR_SCEF...2	fm	wilcox	0.723673609831763			
726	2	0	Blue-negativeR_SCEF...2	fm	wilcox	0.723673609831763			
727	1	0.5	Blue-negativeR_SFL...30	fm	wilcox	0.678746806465678			
728	2	0.25	Blue-negativeR_SFL...30	fm	wilcox	0.678746806465678			
729	1	0.611111111111111	Blue-negativeR_6r...31	fm	wilcox	0.490555029031673			
730	2	0.25	Blue-negativeR_6r...31	fm	wilcox	0.490555029031673			
731	1	0.555555555555556	Blue-negativeR_6v...32	fm	wilcox	0.619610228114867			
732	2	0.75	Blue-negativeR_6v...32	fm	wilcox	0.619610228114867			
733	1	0.166666666666667	Blue-negativeL_Accumbe	fm	wilcox	0.550466854058989			
734	2	0	Blue-negativeL_Accumbe	fm	wilcox	0.550466854058989			
735	1	0.611111111111111	Blue-negativeL_Caudate.	fm	wilcox	0.438429314130547			
736	2	0.25	Blue-negativeL_Caudate.	fm	wilcox	0.438429314130547			
737	1	1.72222222222222	Blue-negativeL_Cerebellu	fm	wilcox	0.384269340713101			
738	2	0.75	Blue-negativeL_Cerebellu	fm	wilcox	0.384269340713101			
739	1	1.05555555555556	Blue-negativeL_Pallidum	fm	wilcox	0.23135382247082			
740	2	0.25	Blue-negativeL_Pallidum	fm	wilcox	0.23135382247082			
741	1	0.722222222222222	Blue-negativeL_Putamen	fm	wilcox	0.122830236892477			
742	2	0	Blue-negativeL_Putamen	fm	wilcox	0.122830236892477			
743	1	0.944444444444444	Blue-negativeL_Thalamus	fm	wilcox	0.728529454946688			
744	2	0.5	Blue-negativeL_Thalamus	fm	wilcox	0.728529454946688			
745	1	0.833333333333333	Blue-negativeL_VentralD(	fm	wilcox	0.163562697973877			
746	2	0	Blue-negativeL_VentralD(	fm	wilcox	0.163562697973877			
747	1	0.222222222222222	Blue-negativeR_Accumb	fm	wilcox	0.339727775866098			
748	2	0	Blue-negativeR_Accumb	fm	wilcox	0.339727775866098			
749	1	0.777777777777778	Blue-negativeR_Caudate	fm	wilcox	0.122830236892477			
750	2	0	Blue-negativeR_Caudate	fm	wilcox	0.122830236892477			
751	1	1.22222222222222	Blue-negativeR_Cerebell	fm	wilcox	0.381925801437231			
752	2	0.5	Blue-negativeR_Cerebell	fm	wilcox	0.381925801437231			
753	1	0.388888888888889	Blue-negativeR_Pallidum	fm	wilcox	0.64972262878551			
754	2	0.5	Blue-negativeR_Pallidum	fm	wilcox	0.64972262878551			
755	1	0.611111111111111	Blue-negativeR_Putamer	fm	wilcox	0.211254703600721			
756	2	0	Blue-negativeR_Putamer	fm	wilcox	0.211254703600721			
757	1	0.444444444444444	Blue-negativeR_Thalamu	fm	wilcox	0.613833914186036			
758	2	0.25	Blue-negativeR_Thalamu	fm	wilcox	0.613833914186036			
759	1	0.666666666666667	Blue-negativeR_VentralD	fm	wilcox	0.120658175870867			
760	2	0	Blue-negativeR_VentralD	fm	wilcox	0.120658175870867			
761	1	5.72222222222222	Blue-negativeL_cortical...	fm	wilcox	0.863896077725539			
762	2	5.25	Blue-negativeL_cortical...	fm	wilcox	0.863896077725539			
763	1	6.94444444444444	Blue-negativeR_cortical...	fm	wilcox	0.93179553472549			
764	2	7	Blue-negativeR_cortical...	fm	wilcox	0.93179553472549			
765	1	5.88888888888889	Blue-negativeL_subcortic	fm	wilcox	0.344541422717149			
766	2	2	Blue-negativeL_subcortic	fm	wilcox	0.344541422717149			
767	1	4.33333333333333	Blue-negativeR_subcortic	fm	wilcox	0.102439545158559			
768	2	1.25	Blue-negativeR_subcortic	fm	wilcox	0.102439545158559			
769	1	2.5	Red-positiveL_1...3	barthel	kruskal	0.356114534767736			
770	2	1	Red-positiveL_1...3	barthel	kruskal	0.356114534767736			
771	3	2.83333333333333	Red-positiveL_1...3	barthel	kruskal	0.356114534767736			
772	4	0.666666666666667	Red-positiveL_1...3	barthel	kruskal	0.356114534767736			
773	5	2.33333333333333	Red-positiveL_1...3	barthel	kruskal	0.356114534767736			
774	1	1.5	Red-positiveL_2...4	barthel	kruskal	0.445170104575394			
775	2	0.4	Red-positiveL_2...4	barthel	kruskal	0.445170104575394			
776	3	0.5	Red-positiveL_2...4	barthel	kruskal	0.445170104575394			
777	4	0.666666666666667	Red-positiveL_2...4	barthel	kruskal	0.445170104575394			
778	5	0.333333333333333	Red-positiveL_2...4	barthel	kruskal	0.445170104575394			

779	1	0	Red-positiveL_3a...5	barthel	kruskal	0.718115249121574			
780	2	0.6	Red-positiveL_3a...5	barthel	kruskal	0.718115249121574			
781	3	0.833333333333333	Red-positiveL_3a...5	barthel	kruskal	0.718115249121574			
782	4	1	Red-positiveL_3a...5	barthel	kruskal	0.718115249121574			
783	5	0.666666666666667	Red-positiveL_3a...5	barthel	kruskal	0.718115249121574			
784	1	0	Red-positiveL_3b...6	barthel	kruskal	0.757256988513783			
785	2	0.4	Red-positiveL_3b...6	barthel	kruskal	0.757256988513783			
786	3	0.333333333333333	Red-positiveL_3b...6	barthel	kruskal	0.757256988513783			
787	4	0.333333333333333	Red-positiveL_3b...6	barthel	kruskal	0.757256988513783			
788	5	0.666666666666667	Red-positiveL_3b...6	barthel	kruskal	0.757256988513783			
789	1	0	Red-positiveL_4...7	barthel	kruskal	0.552262726531441			
790	2	0.2	Red-positiveL_4...7	barthel	kruskal	0.552262726531441			
791	3	0	Red-positiveL_4...7	barthel	kruskal	0.552262726531441			
792	4	0.333333333333333	Red-positiveL_4...7	barthel	kruskal	0.552262726531441			
793	5	0.333333333333333	Red-positiveL_4...7	barthel	kruskal	0.552262726531441			
794	1	0	Red-positiveL_24dd...8	barthel	kruskal	0.55779674536558			
795	2	1.2	Red-positiveL_24dd...8	barthel	kruskal	0.55779674536558			
796	3	0.666666666666667	Red-positiveL_24dd...8	barthel	kruskal	0.55779674536558			
797	4	1	Red-positiveL_24dd...8	barthel	kruskal	0.55779674536558			
798	5	0.833333333333333	Red-positiveL_24dd...8	barthel	kruskal	0.55779674536558			
799	1	0.5	Red-positiveL_24dv...9	barthel	kruskal	0.946488587926551			
800	2	1.4	Red-positiveL_24dv...9	barthel	kruskal	0.946488587926551			
801	3	1	Red-positiveL_24dv...9	barthel	kruskal	0.946488587926551			
802	4	1	Red-positiveL_24dv...9	barthel	kruskal	0.946488587926551			
803	5	2	Red-positiveL_24dv...9	barthel	kruskal	0.946488587926551			
804	1	0.5	Red-positiveL_6a...10	barthel	kruskal	0.834418898551522			
805	2	0.2	Red-positiveL_6a...10	barthel	kruskal	0.834418898551522			
806	3	0.166666666666667	Red-positiveL_6a...10	barthel	kruskal	0.834418898551522			
807	4	1	Red-positiveL_6a...10	barthel	kruskal	0.834418898551522			
808	5	0.166666666666667	Red-positiveL_6a...10	barthel	kruskal	0.834418898551522			
809	1	1.5	Red-positiveL_6d...11	barthel	kruskal	0.162433086800581			
810	2	0.2	Red-positiveL_6d...11	barthel	kruskal	0.162433086800581			
811	3	0.333333333333333	Red-positiveL_6d...11	barthel	kruskal	0.162433086800581			
812	4	1.33333333333333	Red-positiveL_6d...11	barthel	kruskal	0.162433086800581			
813	5	1.5	Red-positiveL_6d...11	barthel	kruskal	0.162433086800581			
814	1	1	Red-positiveL_6ma...12	barthel	kruskal	0.988871008681393			
815	2	0.6	Red-positiveL_6ma...12	barthel	kruskal	0.988871008681393			
816	3	0.666666666666667	Red-positiveL_6ma...12	barthel	kruskal	0.988871008681393			
817	4	0.666666666666667	Red-positiveL_6ma...12	barthel	kruskal	0.988871008681393			
818	5	1	Red-positiveL_6ma...12	barthel	kruskal	0.988871008681393			
819	1	1	Red-positiveL_6mp...13	barthel	kruskal	0.501255816157689			
820	2	0	Red-positiveL_6mp...13	barthel	kruskal	0.501255816157689			
821	3	0.166666666666667	Red-positiveL_6mp...13	barthel	kruskal	0.501255816157689			
822	4	0.333333333333333	Red-positiveL_6mp...13	barthel	kruskal	0.501255816157689			
823	5	0.166666666666667	Red-positiveL_6mp...13	barthel	kruskal	0.501255816157689			
824	1	0	Red-positiveL_SCEF...14	barthel	kruskal	0.842519625226112			
825	2	0.2	Red-positiveL_SCEF...14	barthel	kruskal	0.842519625226112			
826	3	0.166666666666667	Red-positiveL_SCEF...14	barthel	kruskal	0.842519625226112			
827	4	0.333333333333333	Red-positiveL_SCEF...14	barthel	kruskal	0.842519625226112			
828	5	0.5	Red-positiveL_SCEF...14	barthel	kruskal	0.842519625226112			
829	1	1.5	Red-positiveL_SFL...15	barthel	kruskal	0.509407721767467			
830	2	0.6	Red-positiveL_SFL...15	barthel	kruskal	0.509407721767467			
831	3	0	Red-positiveL_SFL...15	barthel	kruskal	0.509407721767467			
832	4	0.333333333333333	Red-positiveL_SFL...15	barthel	kruskal	0.509407721767467			
833	5	0.166666666666667	Red-positiveL_SFL...15	barthel	kruskal	0.509407721767467			
834	1	0	Red-positiveL_6r...16	barthel	kruskal	0.806543757276305			
835	2	0.4	Red-positiveL_6r...16	barthel	kruskal	0.806543757276305			
836	3	0.5	Red-positiveL_6r...16	barthel	kruskal	0.806543757276305			
837	4	0.333333333333333	Red-positiveL_6r...16	barthel	kruskal	0.806543757276305			
838	5	0.333333333333333	Red-positiveL_6r...16	barthel	kruskal	0.806543757276305			

839	1	0	Red-positiveL_6v...17	barthel	kruskal	0.271095119918274			
840	2	0.6	Red-positiveL_6v...17	barthel	kruskal	0.271095119918274			
841	3	2.16666666666667	Red-positiveL_6v...17	barthel	kruskal	0.271095119918274			
842	4	2	Red-positiveL_6v...17	barthel	kruskal	0.271095119918274			
843	5	1.33333333333333	Red-positiveL_6v...17	barthel	kruskal	0.271095119918274			
844	1	1.5	Red-positiveR_1...18	barthel	kruskal	0.974095212610937			
845	2	1.4	Red-positiveR_1...18	barthel	kruskal	0.974095212610937			
846	3	1.66666666666667	Red-positiveR_1...18	barthel	kruskal	0.974095212610937			
847	4	1	Red-positiveR_1...18	barthel	kruskal	0.974095212610937			
848	5	1.5	Red-positiveR_1...18	barthel	kruskal	0.974095212610937			
849	1	0	Red-positiveR_2...19	barthel	kruskal	0.297897682132206			
850	2	0	Red-positiveR_2...19	barthel	kruskal	0.297897682132206			
851	3	0.333333333333333	Red-positiveR_2...19	barthel	kruskal	0.297897682132206			
852	4	0.666666666666667	Red-positiveR_2...19	barthel	kruskal	0.297897682132206			
853	5	0.5	Red-positiveR_2...19	barthel	kruskal	0.297897682132206			
854	1	0	Red-positiveR_3a...20	barthel	kruskal	0.674862962103761			
855	2	0.4	Red-positiveR_3a...20	barthel	kruskal	0.674862962103761			
856	3	0.5	Red-positiveR_3a...20	barthel	kruskal	0.674862962103761			
857	4	0	Red-positiveR_3a...20	barthel	kruskal	0.674862962103761			
858	5	1.16666666666667	Red-positiveR_3a...20	barthel	kruskal	0.674862962103761			
859	1	0.5	Red-positiveR_3b...21	barthel	kruskal	0.803353669564276			
860	2	0.6	Red-positiveR_3b...21	barthel	kruskal	0.803353669564276			
861	3	0.333333333333333	Red-positiveR_3b...21	barthel	kruskal	0.803353669564276			
862	4	0	Red-positiveR_3b...21	barthel	kruskal	0.803353669564276			
863	5	0.833333333333333	Red-positiveR_3b...21	barthel	kruskal	0.803353669564276			
864	1	1.5	Red-positiveR_4...22	barthel	kruskal	0.177675696294938			
865	2	0.2	Red-positiveR_4...22	barthel	kruskal	0.177675696294938			
866	3	0	Red-positiveR_4...22	barthel	kruskal	0.177675696294938			
867	4	0	Red-positiveR_4...22	barthel	kruskal	0.177675696294938			
868	5	0	Red-positiveR_4...22	barthel	kruskal	0.177675696294938			
869	1	2	Red-positiveR_24dd...23	barthel	kruskal	0.267864035295131			
870	2	0.2	Red-positiveR_24dd...23	barthel	kruskal	0.267864035295131			
871	3	0	Red-positiveR_24dd...23	barthel	kruskal	0.267864035295131			
872	4	1	Red-positiveR_24dd...23	barthel	kruskal	0.267864035295131			
873	5	0.833333333333333	Red-positiveR_24dd...23	barthel	kruskal	0.267864035295131			
874	1	0	Red-positiveR_24dv...24	barthel	kruskal	0.805042952935485			
875	2	0.8	Red-positiveR_24dv...24	barthel	kruskal	0.805042952935485			
876	3	0.333333333333333	Red-positiveR_24dv...24	barthel	kruskal	0.805042952935485			
877	4	0.333333333333333	Red-positiveR_24dv...24	barthel	kruskal	0.805042952935485			
878	5	0.5	Red-positiveR_24dv...24	barthel	kruskal	0.805042952935485			
879	1	0	Red-positiveR_6a...25	barthel	kruskal	0.0933234014112658			
880	2	0.6	Red-positiveR_6a...25	barthel	kruskal	0.0933234014112658			
881	3	0.166666666666667	Red-positiveR_6a...25	barthel	kruskal	0.0933234014112658			
882	4	0	Red-positiveR_6a...25	barthel	kruskal	0.0933234014112658			
883	5	0	Red-positiveR_6a...25	barthel	kruskal	0.0933234014112658			
884	1	0	Red-positiveR_6d...26	barthel	kruskal	0.300829812276247			
885	2	0.4	Red-positiveR_6d...26	barthel	kruskal	0.300829812276247			
886	3	0.333333333333333	Red-positiveR_6d...26	barthel	kruskal	0.300829812276247			
887	4	0.333333333333333	Red-positiveR_6d...26	barthel	kruskal	0.300829812276247			
888	5	1.5	Red-positiveR_6d...26	barthel	kruskal	0.300829812276247			
889	1	1	Red-positiveR_6ma...27	barthel	kruskal	0.14253950967963			
890	2	0.4	Red-positiveR_6ma...27	barthel	kruskal	0.14253950967963			
891	3	0.833333333333333	Red-positiveR_6ma...27	barthel	kruskal	0.14253950967963			
892	4	1.66666666666667	Red-positiveR_6ma...27	barthel	kruskal	0.14253950967963			
893	5	0.833333333333333	Red-positiveR_6ma...27	barthel	kruskal	0.14253950967963			
894	1	0	Red-positiveR_6mp...28	barthel	kruskal	0.71094525304213			
895	2	0.2	Red-positiveR_6mp...28	barthel	kruskal	0.71094525304213			
896	3	0	Red-positiveR_6mp...28	barthel	kruskal	0.71094525304213			
897	4	0	Red-positiveR_6mp...28	barthel	kruskal	0.71094525304213			
898	5	0.166666666666667	Red-positiveR_6mp...28	barthel	kruskal	0.71094525304213			

899	1	0	Red-positiveR_SCEF...29	barthel	kruskal	0.0439348401184536			
900	2	0	Red-positiveR_SCEF...29	barthel	kruskal	0.0439348401184536			
901	3	0.666666666666667	Red-positiveR_SCEF...29	barthel	kruskal	0.0439348401184536			
902	4	0	Red-positiveR_SCEF...29	barthel	kruskal	0.0439348401184536			
903	5	1	Red-positiveR_SCEF...29	barthel	kruskal	0.0439348401184536			
904	1	3.5	Red-positiveR_SFL...30	barthel	kruskal	0.645878582482953			
905	2	0.4	Red-positiveR_SFL...30	barthel	kruskal	0.645878582482953			
906	3	0.833333333333333	Red-positiveR_SFL...30	barthel	kruskal	0.645878582482953			
907	4	0	Red-positiveR_SFL...30	barthel	kruskal	0.645878582482953			
908	5	0.666666666666667	Red-positiveR_SFL...30	barthel	kruskal	0.645878582482953			
909	1	0.5	Red-positiveR_6r...31	barthel	kruskal	0.446851381945149			
910	2	0.2	Red-positiveR_6r...31	barthel	kruskal	0.446851381945149			
911	3	0	Red-positiveR_6r...31	barthel	kruskal	0.446851381945149			
912	4	0	Red-positiveR_6r...31	barthel	kruskal	0.446851381945149			
913	5	0.166666666666667	Red-positiveR_6r...31	barthel	kruskal	0.446851381945149			
914	1	0	Red-positiveR_6v...32	barthel	kruskal	0.356187004316263			
915	2	1.8	Red-positiveR_6v...32	barthel	kruskal	0.356187004316263			
916	3	1	Red-positiveR_6v...32	barthel	kruskal	0.356187004316263			
917	4	0.333333333333333	Red-positiveR_6v...32	barthel	kruskal	0.356187004316263			
918	5	1.16666666666667	Red-positiveR_6v...32	barthel	kruskal	0.356187004316263			
919	1	0.5	Red-positiveL_Accumbe	barthel	kruskal	0.464026842876978			
920	2	0	Red-positiveL_Accumbe	barthel	kruskal	0.464026842876978			
921	3	0.5	Red-positiveL_Accumbe	barthel	kruskal	0.464026842876978			
922	4	0	Red-positiveL_Accumbe	barthel	kruskal	0.464026842876978			
923	5	0.666666666666667	Red-positiveL_Accumbe	barthel	kruskal	0.464026842876978			
924	1	1.5	Red-positiveL_Caudate..	barthel	kruskal	0.420230530582228			
925	2	0.6	Red-positiveL_Caudate..	barthel	kruskal	0.420230530582228			
926	3	0.166666666666667	Red-positiveL_Caudate..	barthel	kruskal	0.420230530582228			
927	4	2.33333333333333	Red-positiveL_Caudate..	barthel	kruskal	0.420230530582228			
928	5	0.666666666666667	Red-positiveL_Caudate..	barthel	kruskal	0.420230530582228			
929	1	1	Red-positiveL_Cerebellu	barthel	kruskal	0.35896560948332			
930	2	1.6	Red-positiveL_Cerebellu	barthel	kruskal	0.35896560948332			
931	3	1.66666666666667	Red-positiveL_Cerebellu	barthel	kruskal	0.35896560948332			
932	4	0	Red-positiveL_Cerebellu	barthel	kruskal	0.35896560948332			
933	5	0.833333333333333	Red-positiveL_Cerebellu	barthel	kruskal	0.35896560948332			
934	1	1	Red-positiveL_Pallidum..	barthel	kruskal	0.92456081985157			
935	2	0.4	Red-positiveL_Pallidum..	barthel	kruskal	0.92456081985157			
936	3	0.5	Red-positiveL_Pallidum..	barthel	kruskal	0.92456081985157			
937	4	0.333333333333333	Red-positiveL_Pallidum..	barthel	kruskal	0.92456081985157			
938	5	0.666666666666667	Red-positiveL_Pallidum..	barthel	kruskal	0.92456081985157			
939	1	2	Red-positiveL_Putamen..	barthel	kruskal	0.320257350820885			
940	2	0.8	Red-positiveL_Putamen..	barthel	kruskal	0.320257350820885			
941	3	0.666666666666667	Red-positiveL_Putamen..	barthel	kruskal	0.320257350820885			
942	4	2	Red-positiveL_Putamen..	barthel	kruskal	0.320257350820885			
943	5	0.5	Red-positiveL_Putamen..	barthel	kruskal	0.320257350820885			
944	1	1	Red-positiveL_Thalamus	barthel	kruskal	0.756895075890329			
945	2	0.6	Red-positiveL_Thalamus	barthel	kruskal	0.756895075890329			
946	3	1	Red-positiveL_Thalamus	barthel	kruskal	0.756895075890329			
947	4	0.333333333333333	Red-positiveL_Thalamus	barthel	kruskal	0.756895075890329			
948	5	0.833333333333333	Red-positiveL_Thalamus	barthel	kruskal	0.756895075890329			
949	1	2.5	Red-positiveL_VentralDC	barthel	kruskal	0.436287211035561			
950	2	0.6	Red-positiveL_VentralDC	barthel	kruskal	0.436287211035561			
951	3	0.666666666666667	Red-positiveL_VentralDC	barthel	kruskal	0.436287211035561			
952	4	1.33333333333333	Red-positiveL_VentralDC	barthel	kruskal	0.436287211035561			
953	5	2.66666666666667	Red-positiveL_VentralDC	barthel	kruskal	0.436287211035561			
954	1	0	Red-positiveR_Accumbe	barthel	kruskal	0.308441041184003			
955	2	0	Red-positiveR_Accumbe	barthel	kruskal	0.308441041184003			
956	3	0.333333333333333	Red-positiveR_Accumbe	barthel	kruskal	0.308441041184003			
957	4	0.666666666666667	Red-positiveR_Accumbe	barthel	kruskal	0.308441041184003			
958	5	0	Red-positiveR_Accumbe	barthel	kruskal	0.308441041184003			

959	1	0.5	Red-positiveR_Caudate..	barthel	kruskal	0.92356813578338			
960	2	1.4	Red-positiveR_Caudate..	barthel	kruskal	0.92356813578338			
961	3	0.833333333333333	Red-positiveR_Caudate..	barthel	kruskal	0.92356813578338			
962	4	1	Red-positiveR_Caudate..	barthel	kruskal	0.92356813578338			
963	5	0.833333333333333	Red-positiveR_Caudate..	barthel	kruskal	0.92356813578338			
964	1	0.5	Red-positiveR_Cerebellu	barthel	kruskal	0.991605153010803			
965	2	0.8	Red-positiveR_Cerebellu	barthel	kruskal	0.991605153010803			
966	3	2.33333333333333	Red-positiveR_Cerebellu	barthel	kruskal	0.991605153010803			
967	4	1	Red-positiveR_Cerebellu	barthel	kruskal	0.991605153010803			
968	5	1.33333333333333	Red-positiveR_Cerebellu	barthel	kruskal	0.991605153010803			
969	1	2.5	Red-positiveR_Pallidum..	barthel	kruskal	0.643351913091182			
970	2	0.2	Red-positiveR_Pallidum..	barthel	kruskal	0.643351913091182			
971	3	1	Red-positiveR_Pallidum..	barthel	kruskal	0.643351913091182			
972	4	1.33333333333333	Red-positiveR_Pallidum..	barthel	kruskal	0.643351913091182			
973	5	0.5	Red-positiveR_Pallidum..	barthel	kruskal	0.643351913091182			
974	1	4	Red-positiveR_Putamen.	barthel	kruskal	0.225282067300399			
975	2	1.2	Red-positiveR_Putamen.	barthel	kruskal	0.225282067300399			
976	3	0.833333333333333	Red-positiveR_Putamen.	barthel	kruskal	0.225282067300399			
977	4	1.33333333333333	Red-positiveR_Putamen.	barthel	kruskal	0.225282067300399			
978	5	0.166666666666667	Red-positiveR_Putamen.	barthel	kruskal	0.225282067300399			
979	1	3.5	Red-positiveR_Thalamus	barthel	kruskal	0.0867630992030055			
980	2	1	Red-positiveR_Thalamus	barthel	kruskal	0.0867630992030055			
981	3	0.166666666666667	Red-positiveR_Thalamus	barthel	kruskal	0.0867630992030055			
982	4	0.666666666666667	Red-positiveR_Thalamus	barthel	kruskal	0.0867630992030055			
983	5	0.166666666666667	Red-positiveR_Thalamus	barthel	kruskal	0.0867630992030055			
984	1	0	Red-positiveR_VentralDC	barthel	kruskal	0.564315414865013			
985	2	0.8	Red-positiveR_VentralDC	barthel	kruskal	0.564315414865013			
986	3	1	Red-positiveR_VentralDC	barthel	kruskal	0.564315414865013			
987	4	0.333333333333333	Red-positiveR_VentralDC	barthel	kruskal	0.564315414865013			
988	5	0.333333333333333	Red-positiveR_VentralDC	barthel	kruskal	0.564315414865013			
989	1	10	Red-positiveL_cortical...4	barthel	kruskal	0.609658712924206			
990	2	6.8	Red-positiveL_cortical...4	barthel	kruskal	0.609658712924206			
991	3	9.5	Red-positiveL_cortical...4	barthel	kruskal	0.609658712924206			
992	4	10.3333333333333	Red-positiveL_cortical...4	barthel	kruskal	0.609658712924206			
993	5	13.3333333333333	Red-positiveL_cortical...4	barthel	kruskal	0.609658712924206			
994	1	10.5	Red-positiveR_cortical...4	barthel	kruskal	0.604427258888844			
995	2	7.6	Red-positiveR_cortical...4	barthel	kruskal	0.604427258888844			
996	3	7	Red-positiveR_cortical...4	barthel	kruskal	0.604427258888844			
997	4	5.33333333333333	Red-positiveR_cortical...4	barthel	kruskal	0.604427258888844			
998	5	10.8333333333333	Red-positiveR_cortical...4	barthel	kruskal	0.604427258888844			
999	1	9.5	Red-positiveL_subcortica	barthel	kruskal	0.604559067152819			
100	2	4.6	Red-positiveL_subcortica	barthel	kruskal	0.604559067152819			
100	3	5.66666666666667	Red-positiveL_subcortica	barthel	kruskal	0.604559067152819			
100	4	6.66666666666667	Red-positiveL_subcortica	barthel	kruskal	0.604559067152819			
100	5	6.5	Red-positiveL_subcortica	barthel	kruskal	0.604559067152819			
100	1	11	Red-positiveR_subcortica	barthel	kruskal	0.369033528576556			
100	2	5.4	Red-positiveR_subcortica	barthel	kruskal	0.369033528576556			
100	3	6.5	Red-positiveR_subcortica	barthel	kruskal	0.369033528576556			
100	4	6.33333333333333	Red-positiveR_subcortica	barthel	kruskal	0.369033528576556			
100	5	3.33333333333333	Red-positiveR_subcortica	barthel	kruskal	0.369033528576556			
100	1	2.5	Blue-negativeL_1...3	barthel	kruskal	0.724987606044619			
101	2	0.8	Blue-negativeL_1...3	barthel	kruskal	0.724987606044619			
101	3	1.16666666666667	Blue-negativeL_1...3	barthel	kruskal	0.724987606044619			
101	4	1	Blue-negativeL_1...3	barthel	kruskal	0.724987606044619			
101	5	1	Blue-negativeL_1...3	barthel	kruskal	0.724987606044619			
101	1	1	Blue-negativeL_2...4	barthel	kruskal	0.512274764246083			
101	2	0.2	Blue-negativeL_2...4	barthel	kruskal	0.512274764246083			
101	3	0	Blue-negativeL_2...4	barthel	kruskal	0.512274764246083			
101	4	0.666666666666667	Blue-negativeL_2...4	barthel	kruskal	0.512274764246083			
101	5	0.333333333333333	Blue-negativeL_2...4	barthel	kruskal	0.512274764246083			

101	1	0.5	Blue-negativeL_3a...5	barthel	kruskal	0.115663627608864			
102	2	0	Blue-negativeL_3a...5	barthel	kruskal	0.115663627608864			
102	3	0.333333333333333	Blue-negativeL_3a...5	barthel	kruskal	0.115663627608864			
102	4	0.666666666666667	Blue-negativeL_3a...5	barthel	kruskal	0.115663627608864			
102	5	0	Blue-negativeL_3a...5	barthel	kruskal	0.115663627608864			
102	1	0	Blue-negativeL_3b...6	barthel	kruskal	0.0568710963278566			
102	2	0	Blue-negativeL_3b...6	barthel	kruskal	0.0568710963278566			
102	3	0.166666666666667	Blue-negativeL_3b...6	barthel	kruskal	0.0568710963278566			
102	4	1.33333333333333	Blue-negativeL_3b...6	barthel	kruskal	0.0568710963278566			
102	5	0	Blue-negativeL_3b...6	barthel	kruskal	0.0568710963278566			
102	1	0	Blue-negativeL_4...7	barthel	kruskal	0.42624102731867			
103	2	0	Blue-negativeL_4...7	barthel	kruskal	0.42624102731867			
103	3	0	Blue-negativeL_4...7	barthel	kruskal	0.42624102731867			
103	4	0.666666666666667	Blue-negativeL_4...7	barthel	kruskal	0.42624102731867			
103	5	0.166666666666667	Blue-negativeL_4...7	barthel	kruskal	0.42624102731867			
103	1	0	Blue-negativeL_24dd...8	barthel	kruskal	0.423263967900893			
103	2	0.4	Blue-negativeL_24dd...8	barthel	kruskal	0.423263967900893			
103	3	0.5	Blue-negativeL_24dd...8	barthel	kruskal	0.423263967900893			
103	4	1.66666666666667	Blue-negativeL_24dd...8	barthel	kruskal	0.423263967900893			
103	5	0.333333333333333	Blue-negativeL_24dd...8	barthel	kruskal	0.423263967900893			
103	1	2	Blue-negativeL_24dv...9	barthel	kruskal	0.0642226909628472			
104	2	0.6	Blue-negativeL_24dv...9	barthel	kruskal	0.0642226909628472			
104	3	0.333333333333333	Blue-negativeL_24dv...9	barthel	kruskal	0.0642226909628472			
104	4	1.66666666666667	Blue-negativeL_24dv...9	barthel	kruskal	0.0642226909628472			
104	5	0	Blue-negativeL_24dv...9	barthel	kruskal	0.0642226909628472			
104	1	1	Blue-negativeL_6a...10	barthel	kruskal	0.0673965895648809			
104	2	0.2	Blue-negativeL_6a...10	barthel	kruskal	0.0673965895648809			
104	3	0.5	Blue-negativeL_6a...10	barthel	kruskal	0.0673965895648809			
104	4	0	Blue-negativeL_6a...10	barthel	kruskal	0.0673965895648809			
104	5	0	Blue-negativeL_6a...10	barthel	kruskal	0.0673965895648809			
104	1	0.5	Blue-negativeL_6d...11	barthel	kruskal	0.621990788196283			
105	2	1	Blue-negativeL_6d...11	barthel	kruskal	0.621990788196283			
105	3	0.333333333333333	Blue-negativeL_6d...11	barthel	kruskal	0.621990788196283			
105	4	0.666666666666667	Blue-negativeL_6d...11	barthel	kruskal	0.621990788196283			
105	5	0.166666666666667	Blue-negativeL_6d...11	barthel	kruskal	0.621990788196283			
105	1	0.5	Blue-negativeL_6ma...12	barthel	kruskal	0.347405648928378			
105	2	0	Blue-negativeL_6ma...12	barthel	kruskal	0.347405648928378			
105	3	0.166666666666667	Blue-negativeL_6ma...12	barthel	kruskal	0.347405648928378			
105	4	0.666666666666667	Blue-negativeL_6ma...12	barthel	kruskal	0.347405648928378			
105	5	0.666666666666667	Blue-negativeL_6ma...12	barthel	kruskal	0.347405648928378			
105	1	0	Blue-negativeL_6mp...13	barthel	kruskal	NA			
106	2	0	Blue-negativeL_6mp...13	barthel	kruskal	NA			
106	3	0	Blue-negativeL_6mp...13	barthel	kruskal	NA			
106	4	0	Blue-negativeL_6mp...13	barthel	kruskal	NA			
106	5	0	Blue-negativeL_6mp...13	barthel	kruskal	NA			
106	1	0.5	Blue-negativeL_SCEF...1	barthel	kruskal	0.0856602730713994			
106	2	0	Blue-negativeL_SCEF...1	barthel	kruskal	0.0856602730713994			
106	3	0	Blue-negativeL_SCEF...1	barthel	kruskal	0.0856602730713994			
106	4	0.666666666666667	Blue-negativeL_SCEF...1	barthel	kruskal	0.0856602730713994			
106	5	0.166666666666667	Blue-negativeL_SCEF...1	barthel	kruskal	0.0856602730713994			
106	1	2.5	Blue-negativeL_SFL...15	barthel	kruskal	0.597181176085485			
107	2	1	Blue-negativeL_SFL...15	barthel	kruskal	0.597181176085485			
107	3	0.5	Blue-negativeL_SFL...15	barthel	kruskal	0.597181176085485			
107	4	1	Blue-negativeL_SFL...15	barthel	kruskal	0.597181176085485			
107	5	0.5	Blue-negativeL_SFL...15	barthel	kruskal	0.597181176085485			
107	1	0.5	Blue-negativeL_6r...16	barthel	kruskal	0.577702419867387			
107	2	0	Blue-negativeL_6r...16	barthel	kruskal	0.577702419867387			
107	3	0.166666666666667	Blue-negativeL_6r...16	barthel	kruskal	0.577702419867387			
107	4	0.333333333333333	Blue-negativeL_6r...16	barthel	kruskal	0.577702419867387			
107	5	0.333333333333333	Blue-negativeL_6r...16	barthel	kruskal	0.577702419867387			

107	1	0.5	Blue-negativeL_6v...17	barthel	kruskal	0.447433710597506			
108	2	0.6	Blue-negativeL_6v...17	barthel	kruskal	0.447433710597506			
108	3	0.166666666666667	Blue-negativeL_6v...17	barthel	kruskal	0.447433710597506			
108	4	1	Blue-negativeL_6v...17	barthel	kruskal	0.447433710597506			
108	5	0.333333333333333	Blue-negativeL_6v...17	barthel	kruskal	0.447433710597506			
108	1	3	Blue-negativeR_1...18	barthel	kruskal	0.184211593786893			
108	2	1.2	Blue-negativeR_1...18	barthel	kruskal	0.184211593786893			
108	3	0.833333333333333	Blue-negativeR_1...18	barthel	kruskal	0.184211593786893			
108	4	1	Blue-negativeR_1...18	barthel	kruskal	0.184211593786893			
108	5	0.666666666666667	Blue-negativeR_1...18	barthel	kruskal	0.184211593786893			
108	1	2	Blue-negativeR_2...19	barthel	kruskal	0.259482468579559			
109	2	0	Blue-negativeR_2...19	barthel	kruskal	0.259482468579559			
109	3	0	Blue-negativeR_2...19	barthel	kruskal	0.259482468579559			
109	4	0.333333333333333	Blue-negativeR_2...19	barthel	kruskal	0.259482468579559			
109	5	0.333333333333333	Blue-negativeR_2...19	barthel	kruskal	0.259482468579559			
109	1	0.5	Blue-negativeR_3a...20	barthel	kruskal	0.773020416209537			
109	2	0.6	Blue-negativeR_3a...20	barthel	kruskal	0.773020416209537			
109	3	0.166666666666667	Blue-negativeR_3a...20	barthel	kruskal	0.773020416209537			
109	4	0.333333333333333	Blue-negativeR_3a...20	barthel	kruskal	0.773020416209537			
109	5	0.166666666666667	Blue-negativeR_3a...20	barthel	kruskal	0.773020416209537			
109	1	1	Blue-negativeR_3b...21	barthel	kruskal	0.507561823008497			
110	2	0.6	Blue-negativeR_3b...21	barthel	kruskal	0.507561823008497			
110	3	0	Blue-negativeR_3b...21	barthel	kruskal	0.507561823008497			
110	4	0.333333333333333	Blue-negativeR_3b...21	barthel	kruskal	0.507561823008497			
110	5	0.666666666666667	Blue-negativeR_3b...21	barthel	kruskal	0.507561823008497			
110	1	0.5	Blue-negativeR_4...22	barthel	kruskal	0.240646051864433			
110	2	0.6	Blue-negativeR_4...22	barthel	kruskal	0.240646051864433			
110	3	0.166666666666667	Blue-negativeR_4...22	barthel	kruskal	0.240646051864433			
110	4	1	Blue-negativeR_4...22	barthel	kruskal	0.240646051864433			
110	5	0	Blue-negativeR_4...22	barthel	kruskal	0.240646051864433			
110	1	2	Blue-negativeR_24dd...2	barthel	kruskal	0.0237507221888208			
111	2	0.6	Blue-negativeR_24dd...2	barthel	kruskal	0.0237507221888208			
111	3	0.166666666666667	Blue-negativeR_24dd...2	barthel	kruskal	0.0237507221888208			
111	4	2.33333333333333	Blue-negativeR_24dd...2	barthel	kruskal	0.0237507221888208			
111	5	0.333333333333333	Blue-negativeR_24dd...2	barthel	kruskal	0.0237507221888208			
111	1	0	Blue-negativeR_24dv...24	barthel	kruskal	0.63273918628708			
111	2	0.6	Blue-negativeR_24dv...24	barthel	kruskal	0.63273918628708			
111	3	0.333333333333333	Blue-negativeR_24dv...24	barthel	kruskal	0.63273918628708			
111	4	0.333333333333333	Blue-negativeR_24dv...24	barthel	kruskal	0.63273918628708			
111	5	0.333333333333333	Blue-negativeR_24dv...24	barthel	kruskal	0.63273918628708			
111	1	1	Blue-negativeR_6a...25	barthel	kruskal	0.374021791528805			
112	2	0.4	Blue-negativeR_6a...25	barthel	kruskal	0.374021791528805			
112	3	0.333333333333333	Blue-negativeR_6a...25	barthel	kruskal	0.374021791528805			
112	4	0.333333333333333	Blue-negativeR_6a...25	barthel	kruskal	0.374021791528805			
112	5	0.166666666666667	Blue-negativeR_6a...25	barthel	kruskal	0.374021791528805			
112	1	0	Blue-negativeR_6d...26	barthel	kruskal	0.578166913707404			
112	2	0	Blue-negativeR_6d...26	barthel	kruskal	0.578166913707404			
112	3	0.333333333333333	Blue-negativeR_6d...26	barthel	kruskal	0.578166913707404			
112	4	0.333333333333333	Blue-negativeR_6d...26	barthel	kruskal	0.578166913707404			
112	5	0.666666666666667	Blue-negativeR_6d...26	barthel	kruskal	0.578166913707404			
112	1	3	Blue-negativeR_6ma...27	barthel	kruskal	0.126349626287363			
113	2	0.8	Blue-negativeR_6ma...27	barthel	kruskal	0.126349626287363			
113	3	0.666666666666667	Blue-negativeR_6ma...27	barthel	kruskal	0.126349626287363			
113	4	1.33333333333333	Blue-negativeR_6ma...27	barthel	kruskal	0.126349626287363			
113	5	0.166666666666667	Blue-negativeR_6ma...27	barthel	kruskal	0.126349626287363			
113	1	0	Blue-negativeR_6mp...28	barthel	kruskal	0.493245514942397			
113	2	0.2	Blue-negativeR_6mp...28	barthel	kruskal	0.493245514942397			
113	3	0	Blue-negativeR_6mp...28	barthel	kruskal	0.493245514942397			
113	4	0	Blue-negativeR_6mp...28	barthel	kruskal	0.493245514942397			
113	5	0	Blue-negativeR_6mp...28	barthel	kruskal	0.493245514942397			

113	1	1	Blue-negativeR_SCEF...2	barthel	kruskal	0.0404276819945132			
114	2	0	Blue-negativeR_SCEF...2	barthel	kruskal	0.0404276819945132			
114	3	0	Blue-negativeR_SCEF...2	barthel	kruskal	0.0404276819945132			
114	4	0	Blue-negativeR_SCEF...2	barthel	kruskal	0.0404276819945132			
114	5	0	Blue-negativeR_SCEF...2	barthel	kruskal	0.0404276819945132			
114	1	1.5	Blue-negativeR_SFL...30	barthel	kruskal	0.231476825673825			
114	2	0.2	Blue-negativeR_SFL...30	barthel	kruskal	0.231476825673825			
114	3	0.166666666666667	Blue-negativeR_SFL...30	barthel	kruskal	0.231476825673825			
114	4	0.333333333333333	Blue-negativeR_SFL...30	barthel	kruskal	0.231476825673825			
114	5	0.666666666666667	Blue-negativeR_SFL...30	barthel	kruskal	0.231476825673825			
114	1	2.5	Blue-negativeR_6r...31	barthel	kruskal	0.0807982047958454			
115	2	0.6	Blue-negativeR_6r...31	barthel	kruskal	0.0807982047958454			
115	3	0.166666666666667	Blue-negativeR_6r...31	barthel	kruskal	0.0807982047958454			
115	4	0	Blue-negativeR_6r...31	barthel	kruskal	0.0807982047958454			
115	5	0.5	Blue-negativeR_6r...31	barthel	kruskal	0.0807982047958454			
115	1	0.5	Blue-negativeR_6v...32	barthel	kruskal	0.894826270186635			
115	2	0.2	Blue-negativeR_6v...32	barthel	kruskal	0.894826270186635			
115	3	0.666666666666667	Blue-negativeR_6v...32	barthel	kruskal	0.894826270186635			
115	4	1	Blue-negativeR_6v...32	barthel	kruskal	0.894826270186635			
115	5	0.666666666666667	Blue-negativeR_6v...32	barthel	kruskal	0.894826270186635			
115	1	0	Blue-negativeL_Accumbe	barthel	kruskal	0.78089246615514			
116	2	0	Blue-negativeL_Accumbe	barthel	kruskal	0.78089246615514			
116	3	0.166666666666667	Blue-negativeL_Accumbe	barthel	kruskal	0.78089246615514			
116	4	0	Blue-negativeL_Accumbe	barthel	kruskal	0.78089246615514			
116	5	0.333333333333333	Blue-negativeL_Accumbe	barthel	kruskal	0.78089246615514			
116	1	1.5	Blue-negativeL_Caudate.	barthel	kruskal	0.222866389308929			
116	2	0.8	Blue-negativeL_Caudate.	barthel	kruskal	0.222866389308929			
116	3	0.166666666666667	Blue-negativeL_Caudate.	barthel	kruskal	0.222866389308929			
116	4	0.333333333333333	Blue-negativeL_Caudate.	barthel	kruskal	0.222866389308929			
116	5	0.5	Blue-negativeL_Caudate.	barthel	kruskal	0.222866389308929			
116	1	3	Blue-negativeL_Cerebellu	barthel	kruskal	0.853521789723513			
117	2	0.6	Blue-negativeL_Cerebellu	barthel	kruskal	0.853521789723513			
117	3	1.5	Blue-negativeL_Cerebellu	barthel	kruskal	0.853521789723513			
117	4	1	Blue-negativeL_Cerebellu	barthel	kruskal	0.853521789723513			
117	5	2.16666666666667	Blue-negativeL_Cerebellu	barthel	kruskal	0.853521789723513			
117	1	1	Blue-negativeL_Pallidum	barthel	kruskal	0.0161067807540646			
117	2	1	Blue-negativeL_Pallidum	barthel	kruskal	0.0161067807540646			
117	3	0	Blue-negativeL_Pallidum	barthel	kruskal	0.0161067807540646			
117	4	3	Blue-negativeL_Pallidum	barthel	kruskal	0.0161067807540646			
117	5	0.666666666666667	Blue-negativeL_Pallidum	barthel	kruskal	0.0161067807540646			
117	1	0.5	Blue-negativeL_Putamen	barthel	kruskal	0.729346170834161			
118	2	0.4	Blue-negativeL_Putamen	barthel	kruskal	0.729346170834161			
118	3	0.5	Blue-negativeL_Putamen	barthel	kruskal	0.729346170834161			
118	4	1.33333333333333	Blue-negativeL_Putamen	barthel	kruskal	0.729346170834161			
118	5	0.5	Blue-negativeL_Putamen	barthel	kruskal	0.729346170834161			
118	1	3.5	Blue-negativeL_Thalamus	barthel	kruskal	0.0274530659446478			
118	2	1	Blue-negativeL_Thalamus	barthel	kruskal	0.0274530659446478			
118	3	0	Blue-negativeL_Thalamus	barthel	kruskal	0.0274530659446478			
118	4	0.333333333333333	Blue-negativeL_Thalamus	barthel	kruskal	0.0274530659446478			
118	5	1	Blue-negativeL_Thalamus	barthel	kruskal	0.0274530659446478			
118	1	1.5	Blue-negativeL_VentralD(	barthel	kruskal	0.498982223816638			
119	2	0.8	Blue-negativeL_VentralD(	barthel	kruskal	0.498982223816638			
119	3	0.333333333333333	Blue-negativeL_VentralD(	barthel	kruskal	0.498982223816638			
119	4	0	Blue-negativeL_VentralD(	barthel	kruskal	0.498982223816638			
119	5	1	Blue-negativeL_VentralD(	barthel	kruskal	0.498982223816638			
119	1	0	Blue-negativeR_Accumbe	barthel	kruskal	0.588480939047005			
119	2	0	Blue-negativeR_Accumbe	barthel	kruskal	0.588480939047005			
119	3	0.333333333333333	Blue-negativeR_Accumbe	barthel	kruskal	0.588480939047005			
119	4	0.333333333333333	Blue-negativeR_Accumbe	barthel	kruskal	0.588480939047005			
119	5	0.166666666666667	Blue-negativeR_Accumbe	barthel	kruskal	0.588480939047005			

119	1	0.5	Blue-negativeR_Caudate	barthel	kruskal	0.0417031401020217
120	2	0.4	Blue-negativeR_Caudate	barthel	kruskal	0.0417031401020217
120	3	0	Blue-negativeR_Caudate	barthel	kruskal	0.0417031401020217
120	4	2.66666666666667	Blue-negativeR_Caudate	barthel	kruskal	0.0417031401020217
120	5	0.5	Blue-negativeR_Caudate	barthel	kruskal	0.0417031401020217
120	1	3.5	Blue-negativeR_Cerebell	barthel	kruskal	0.228611645758686
120	2	0.4	Blue-negativeR_Cerebell	barthel	kruskal	0.228611645758686
120	3	0.666666666666667	Blue-negativeR_Cerebell	barthel	kruskal	0.228611645758686
120	4	1.66666666666667	Blue-negativeR_Cerebell	barthel	kruskal	0.228611645758686
120	5	1	Blue-negativeR_Cerebell	barthel	kruskal	0.228611645758686
120	1	1	Blue-negativeR_Pallidum	barthel	kruskal	0.61388893532598
121	2	0.4	Blue-negativeR_Pallidum	barthel	kruskal	0.61388893532598
121	3	0.333333333333333	Blue-negativeR_Pallidum	barthel	kruskal	0.61388893532598
121	4	0.666666666666667	Blue-negativeR_Pallidum	barthel	kruskal	0.61388893532598
121	5	0.166666666666667	Blue-negativeR_Pallidum	barthel	kruskal	0.61388893532598
121	1	1	Blue-negativeR_Putamer	barthel	kruskal	0.224869208709799
121	2	1	Blue-negativeR_Putamer	barthel	kruskal	0.224869208709799
121	3	0	Blue-negativeR_Putamer	barthel	kruskal	0.224869208709799
121	4	1	Blue-negativeR_Putamer	barthel	kruskal	0.224869208709799
121	5	0.166666666666667	Blue-negativeR_Putamer	barthel	kruskal	0.224869208709799
121	1	0.5	Blue-negativeR_Thalamu	barthel	kruskal	0.321358813151893
122	2	0.6	Blue-negativeR_Thalamu	barthel	kruskal	0.321358813151893
122	3	0	Blue-negativeR_Thalamu	barthel	kruskal	0.321358813151893
122	4	0.666666666666667	Blue-negativeR_Thalamu	barthel	kruskal	0.321358813151893
122	5	0.5	Blue-negativeR_Thalamu	barthel	kruskal	0.321358813151893
122	1	0	Blue-negativeR_VentralD	barthel	kruskal	0.573847218735313
122	2	0.8	Blue-negativeR_VentralD	barthel	kruskal	0.573847218735313
122	3	0.333333333333333	Blue-negativeR_VentralD	barthel	kruskal	0.573847218735313
122	4	1.33333333333333	Blue-negativeR_VentralD	barthel	kruskal	0.573847218735313
122	5	0.333333333333333	Blue-negativeR_VentralD	barthel	kruskal	0.573847218735313
122	1	12	Blue-negativeL_cortical...	barthel	kruskal	0.0514183724868238
123	2	4.4	Blue-negativeL_cortical...	barthel	kruskal	0.0514183724868238
123	3	3.83333333333333	Blue-negativeL_cortical...	barthel	kruskal	0.0514183724868238
123	4	10	Blue-negativeL_cortical...	barthel	kruskal	0.0514183724868238
123	5	4.16666666666667	Blue-negativeL_cortical...	barthel	kruskal	0.0514183724868238
123	1	18.5	Blue-negativeR_cortical...	barthel	kruskal	0.0933184358657904
123	2	6.6	Blue-negativeR_cortical...	barthel	kruskal	0.0933184358657904
123	3	4	Blue-negativeR_cortical...	barthel	kruskal	0.0933184358657904
123	4	9	Blue-negativeR_cortical...	barthel	kruskal	0.0933184358657904
123	5	5.33333333333333	Blue-negativeR_cortical...	barthel	kruskal	0.0933184358657904
123	1	11	Blue-negativeL_subcortic	barthel	kruskal	0.431883803192443
124	2	3.8	Blue-negativeL_subcortic	barthel	kruskal	0.431883803192443
124	3	2.83333333333333	Blue-negativeL_subcortic	barthel	kruskal	0.431883803192443
124	4	6	Blue-negativeL_subcortic	barthel	kruskal	0.431883803192443
124	5	6.33333333333333	Blue-negativeL_subcortic	barthel	kruskal	0.431883803192443
124	1	6.5	Blue-negativeR_subcortic	barthel	kruskal	0.225998558895447
124	2	3.6	Blue-negativeR_subcortic	barthel	kruskal	0.225998558895447
124	3	1.66666666666667	Blue-negativeR_subcortic	barthel	kruskal	0.225998558895447
124	4	8.33333333333333	Blue-negativeR_subcortic	barthel	kruskal	0.225998558895447
124	5	2.83333333333333	Blue-negativeR_subcortic	barthel	kruskal	0.225998558895447
124	1	2	Red-positiveL_1...3	wolf	wilcox	0.790583927193667
125	2	1.83333333333333	Red-positiveL_1...3	wolf	wilcox	0.790583927193667
125	1	0.5625	Red-positiveL_2...4	wolf	wilcox	0.897146039245118
125	2	0.5	Red-positiveL_2...4	wolf	wilcox	0.897146039245118
125	1	0.6875	Red-positiveL_3a...5	wolf	wilcox	0.802517566326364
125	2	0.666666666666667	Red-positiveL_3a...5	wolf	wilcox	0.802517566326364
125	1	0.4375	Red-positiveL_3b...6	wolf	wilcox	0.827038122216554
125	2	0.333333333333333	Red-positiveL_3b...6	wolf	wilcox	0.827038122216554
125	1	0.1875	Red-positiveL_4...7	wolf	wilcox	0.956043006882769
125	2	0.166666666666667	Red-positiveL_4...7	wolf	wilcox	0.956043006882769

125	1	1.0625	Red-positiveL_24dd...8	wolf	wilcox	0.08818062118211			
126	2	0.166666666666667	Red-positiveL_24dd...8	wolf	wilcox	0.08818062118211			
126	1	1.5	Red-positiveL_24dv...9	wolf	wilcox	0.755487559099565			
126	2	0.833333333333333	Red-positiveL_24dv...9	wolf	wilcox	0.755487559099565			
126	1	0.1875	Red-positiveL_6a...10	wolf	wilcox	0.419451371323936			
126	2	0.666666666666667	Red-positiveL_6a...10	wolf	wilcox	0.419451371323936			
126	1	1.125	Red-positiveL_6d...11	wolf	wilcox	0.0554303300660503			
126	2	0.166666666666667	Red-positiveL_6d...11	wolf	wilcox	0.0554303300660503			
126	1	1	Red-positiveL_6ma...12	wolf	wilcox	0.13459466476528			
126	2	0.166666666666667	Red-positiveL_6ma...12	wolf	wilcox	0.13459466476528			
126	1	0.25	Red-positiveL_6mp...13	wolf	wilcox	0.912548622816626			
127	2	0.166666666666667	Red-positiveL_6mp...13	wolf	wilcox	0.912548622816626			
127	1	0.25	Red-positiveL_SCEF...14	wolf	wilcox	0.578847307258135			
127	2	0.333333333333333	Red-positiveL_SCEF...14	wolf	wilcox	0.578847307258135			
127	1	0.5	Red-positiveL_SFL...15	wolf	wilcox	0.207164361165831			
127	2	0	Red-positiveL_SFL...15	wolf	wilcox	0.207164361165831			
127	1	0.3125	Red-positiveL_6r...16	wolf	wilcox	0.452471554600455			
127	2	0.5	Red-positiveL_6r...16	wolf	wilcox	0.452471554600455			
127	1	1.25	Red-positiveL_6v...17	wolf	wilcox	0.298516201017352			
127	2	1.66666666666667	Red-positiveL_6v...17	wolf	wilcox	0.298516201017352			
127	1	1.3125	Red-positiveR_1...18	wolf	wilcox	0.878483777018341			
128	2	1.83333333333333	Red-positiveR_1...18	wolf	wilcox	0.878483777018341			
128	1	0.375	Red-positiveR_2...19	wolf	wilcox	0.869394343205162			
128	2	0.166666666666667	Red-positiveR_2...19	wolf	wilcox	0.869394343205162			
128	1	0.6875	Red-positiveR_3a...20	wolf	wilcox	0.451574301379772			
128	2	0.166666666666667	Red-positiveR_3a...20	wolf	wilcox	0.451574301379772			
128	1	0.5625	Red-positiveR_3b...21	wolf	wilcox	0.887443468242707			
128	2	0.333333333333333	Red-positiveR_3b...21	wolf	wilcox	0.887443468242707			
128	1	0.0625	Red-positiveR_4...22	wolf	wilcox	0.460106768888285			
128	2	0.5	Red-positiveR_4...22	wolf	wilcox	0.460106768888285			
128	1	0.75	Red-positiveR_24dd...23	wolf	wilcox	0.42422792308174			
129	2	0.166666666666667	Red-positiveR_24dd...23	wolf	wilcox	0.42422792308174			
129	1	0.4375	Red-positiveR_24dv...24	wolf	wilcox	0.540666674412377			
129	2	0.5	Red-positiveR_24dv...24	wolf	wilcox	0.540666674412377			
129	1	0.25	Red-positiveR_6a...25	wolf	wilcox	0.204885157974755			
129	2	0	Red-positiveR_6a...25	wolf	wilcox	0.204885157974755			
129	1	0.75	Red-positiveR_6d...26	wolf	wilcox	0.529735560654555			
129	2	0.333333333333333	Red-positiveR_6d...26	wolf	wilcox	0.529735560654555			
129	1	0.8125	Red-positiveR_6ma...27	wolf	wilcox	0.264941022704659			
129	2	1	Red-positiveR_6ma...27	wolf	wilcox	0.264941022704659			
129	1	0.125	Red-positiveR_6mp...28	wolf	wilcox	0.415953814916205			
130	2	0	Red-positiveR_6mp...28	wolf	wilcox	0.415953814916205			
130	1	0.5	Red-positiveR_SCEF...29	wolf	wilcox	0.761506327066851			
130	2	0.333333333333333	Red-positiveR_SCEF...29	wolf	wilcox	0.761506327066851			
130	1	0.5625	Red-positiveR_SFL...30	wolf	wilcox	0.637069868947077			
130	2	1.5	Red-positiveR_SFL...30	wolf	wilcox	0.637069868947077			
130	1	0.1875	Red-positiveR_6r...31	wolf	wilcox	0.292277481397128			
130	2	0	Red-positiveR_6r...31	wolf	wilcox	0.292277481397128			
130	1	1.125	Red-positiveR_6v...32	wolf	wilcox	0.552325365254726			
130	2	0.833333333333333	Red-positiveR_6v...32	wolf	wilcox	0.552325365254726			
130	1	0.375	Red-positiveL_Accumbei	wolf	wilcox	0.801422131919437			
131	2	0.333333333333333	Red-positiveL_Accumbei	wolf	wilcox	0.801422131919437			
131	1	0.75	Red-positiveL_Caudate..	wolf	wilcox	0.323799432939694			
131	2	1	Red-positiveL_Caudate..	wolf	wilcox	0.323799432939694			
131	1	1.3125	Red-positiveL_Cerebellur	wolf	wilcox	0.361233792063812			
131	2	0.666666666666667	Red-positiveL_Cerebellur	wolf	wilcox	0.361233792063812			
131	1	0.5625	Red-positiveL_Pallidum..	wolf	wilcox	0.709058918935872			
131	2	0.5	Red-positiveL_Pallidum..	wolf	wilcox	0.709058918935872			
131	1	0.875	Red-positiveL_Putamen..	wolf	wilcox	0.873591364476431			
131	2	1.16666666666667	Red-positiveL_Putamen..	wolf	wilcox	0.873591364476431			

131	1	0.75	Red-positiveL_Thalamus.	wolf	wilcox	0.66015872435694
132	2	0.833333333333333	Red-positiveL_Thalamus.	wolf	wilcox	0.66015872435694
132	1	1.8125	Red-positiveL_VentralDC	wolf	wilcox	0.276453866420421
132	2	0.5	Red-positiveL_VentralDC	wolf	wilcox	0.276453866420421
132	1	0.125	Red-positiveR_Accumbe	wolf	wilcox	0.155189453296383
132	2	0.333333333333333	Red-positiveR_Accumbe	wolf	wilcox	0.155189453296383
132	1	0.8125	Red-positiveR_Caudate..	wolf	wilcox	0.292833652646016
132	2	1.33333333333333	Red-positiveR_Caudate..	wolf	wilcox	0.292833652646016
132	1	1.3125	Red-positiveR_Cerebellu	wolf	wilcox	0.868336345940331
132	2	1.5	Red-positiveR_Cerebellu	wolf	wilcox	0.868336345940331
132	1	0.8125	Red-positiveR_Pallidum..	wolf	wilcox	0.421382887757932
133	2	1	Red-positiveR_Pallidum..	wolf	wilcox	0.421382887757932
133	1	1.375	Red-positiveR_Putamen.	wolf	wilcox	0.348620467072946
133	2	0.333333333333333	Red-positiveR_Putamen.	wolf	wilcox	0.348620467072946
133	1	0.75	Red-positiveR_Thalamus	wolf	wilcox	1
133	2	0.666666666666667	Red-positiveR_Thalamus	wolf	wilcox	1
133	1	0.4375	Red-positiveR_VentralDC	wolf	wilcox	0.378981952385132
133	2	1	Red-positiveR_VentralDC	wolf	wilcox	0.378981952385132
133	1	11.25	Red-positiveL_cortical...	wolf	wilcox	0.263759103086457
133	2	7	Red-positiveL_cortical...	wolf	wilcox	0.263759103086457
133	1	8.5	Red-positiveR_cortical...	wolf	wilcox	0.911549420362014
134	2	7.66666666666667	Red-positiveR_cortical...	wolf	wilcox	0.911549420362014
134	1	6.375	Red-positiveL_subcortica	wolf	wilcox	0.85316006061379
134	2	5.5	Red-positiveL_subcortica	wolf	wilcox	0.85316006061379
134	1	5.625	Red-positiveR_subcortica	wolf	wilcox	0.28221860413447
134	2	6.16666666666667	Red-positiveR_subcortica	wolf	wilcox	0.28221860413447
134	1	1.1875	Blue-negativeL_1...3	wolf	wilcox	0.58776553989993
134	2	1	Blue-negativeL_1...3	wolf	wilcox	0.58776553989993
134	1	0.4375	Blue-negativeL_2...4	wolf	wilcox	0.206596063831261
134	2	0	Blue-negativeL_2...4	wolf	wilcox	0.206596063831261
134	1	0.125	Blue-negativeL_3a...5	wolf	wilcox	0.0758073159117505
135	2	0.5	Blue-negativeL_3a...5	wolf	wilcox	0.0758073159117505
135	1	0.125	Blue-negativeL_3b...6	wolf	wilcox	0.757315235713546
135	2	0.5	Blue-negativeL_3b...6	wolf	wilcox	0.757315235713546
135	1	0.0625	Blue-negativeL_4...7	wolf	wilcox	0.460106768888285
135	2	0.333333333333333	Blue-negativeL_4...7	wolf	wilcox	0.460106768888285
135	1	0.625	Blue-negativeL_24dd...8	wolf	wilcox	0.540959173920324
135	2	0.333333333333333	Blue-negativeL_24dd...8	wolf	wilcox	0.540959173920324
135	1	0.3125	Blue-negativeL_24dv...9	wolf	wilcox	0.128834817994108
135	2	1.5	Blue-negativeL_24dv...9	wolf	wilcox	0.128834817994108
135	1	0.1875	Blue-negativeL_6a...10	wolf	wilcox	0.419451371323936
136	2	0.5	Blue-negativeL_6a...10	wolf	wilcox	0.419451371323936
136	1	0.5625	Blue-negativeL_6d...11	wolf	wilcox	0.761596611857917
136	2	0.333333333333333	Blue-negativeL_6d...11	wolf	wilcox	0.761596611857917
136	1	0.375	Blue-negativeL_6ma...12	wolf	wilcox	0.887391718004304
136	2	0.333333333333333	Blue-negativeL_6ma...12	wolf	wilcox	0.887391718004304
136	1	0	Blue-negativeL_6mp...13	wolf	wilcox	NA
136	2	0	Blue-negativeL_6mp...13	wolf	wilcox	NA
136	1	0.1875	Blue-negativeL_SCEF...1	wolf	wilcox	0.956043006882769
136	2	0.166666666666667	Blue-negativeL_SCEF...1	wolf	wilcox	0.956043006882769
136	1	1	Blue-negativeL_SFL...15	wolf	wilcox	0.420224428601178
137	2	0.5	Blue-negativeL_SFL...15	wolf	wilcox	0.420224428601178
137	1	0.25	Blue-negativeL_6r...16	wolf	wilcox	0.722507365624512
137	2	0.166666666666667	Blue-negativeL_6r...16	wolf	wilcox	0.722507365624512
137	1	0.375	Blue-negativeL_6v...17	wolf	wilcox	0.46845063131748
137	2	0.666666666666667	Blue-negativeL_6v...17	wolf	wilcox	0.46845063131748
137	1	1.0625	Blue-negativeR_1...18	wolf	wilcox	0.608619765788113
137	2	1.16666666666667	Blue-negativeR_1...18	wolf	wilcox	0.608619765788113
137	1	0.1875	Blue-negativeR_2...19	wolf	wilcox	1
137	2	0.666666666666667	Blue-negativeR_2...19	wolf	wilcox	1

137	1	0.4375	Blue-negativeR_3a...20	wolf	wilcox	0.0971178734696414			
138	2	0	Blue-negativeR_3a...20	wolf	wilcox	0.0971178734696414			
138	1	0.625	Blue-negativeR_3b...21	wolf	wilcox	0.145058626507254			
138	2	0	Blue-negativeR_3b...21	wolf	wilcox	0.145058626507254			
138	1	0.4375	Blue-negativeR_4...22	wolf	wilcox	0.650755673589828			
138	2	0.166666666666667	Blue-negativeR_4...22	wolf	wilcox	0.650755673589828			
138	1	0.8125	Blue-negativeR_24dd...2	wolf	wilcox	0.516412268396038			
138	2	0.666666666666667	Blue-negativeR_24dd...2	wolf	wilcox	0.516412268396038			
138	1	0.375	Blue-negativeR_24dv...24	wolf	wilcox	0.556502539944398			
138	2	0.333333333333333	Blue-negativeR_24dv...24	wolf	wilcox	0.556502539944398			
138	1	0.3125	Blue-negativeR_6a...25	wolf	wilcox	0.365633042135195			
139	2	0.5	Blue-negativeR_6a...25	wolf	wilcox	0.365633042135195			
139	1	0.3125	Blue-negativeR_6d...26	wolf	wilcox	0.578847307258135			
139	2	0.333333333333333	Blue-negativeR_6d...26	wolf	wilcox	0.578847307258135			
139	1	0.5625	Blue-negativeR_6ma...27	wolf	wilcox	0.11372747010875			
139	2	1.66666666666667	Blue-negativeR_6ma...27	wolf	wilcox	0.11372747010875			
139	1	0.0625	Blue-negativeR_6mp...28	wolf	wilcox	0.609834043673459			
139	2	0	Blue-negativeR_6mp...28	wolf	wilcox	0.609834043673459			
139	1	0.125	Blue-negativeR_SCEF...2	wolf	wilcox	0.609834043673459			
139	2	0	Blue-negativeR_SCEF...2	wolf	wilcox	0.609834043673459			
139	1	0.5	Blue-negativeR_SFL...30	wolf	wilcox	0.893001757860739			
140	2	0.333333333333333	Blue-negativeR_SFL...30	wolf	wilcox	0.893001757860739			
140	1	0.6875	Blue-negativeR_6r...31	wolf	wilcox	0.172344160274776			
140	2	0.166666666666667	Blue-negativeR_6r...31	wolf	wilcox	0.172344160274776			
140	1	0.625	Blue-negativeR_6v...32	wolf	wilcox	0.863469773950528			
140	2	0.5	Blue-negativeR_6v...32	wolf	wilcox	0.863469773950528			
140	1	0.1875	Blue-negativeL_Accumbe	wolf	wilcox	0.416482901999859			
140	2	0	Blue-negativeL_Accumbe	wolf	wilcox	0.416482901999859			
140	1	0.6875	Blue-negativeL_Caudate.	wolf	wilcox	0.15388093243499			
140	2	0.166666666666667	Blue-negativeL_Caudate.	wolf	wilcox	0.15388093243499			
140	1	1.875	Blue-negativeL_Cerebellu	wolf	wilcox	0.321472748709352			
141	2	0.666666666666667	Blue-negativeL_Cerebellu	wolf	wilcox	0.321472748709352			
141	1	1.0625	Blue-negativeL_Pallidum	wolf	wilcox	0.359180329489441			
141	2	0.5	Blue-negativeL_Pallidum	wolf	wilcox	0.359180329489441			
141	1	0.625	Blue-negativeL_Putamen	wolf	wilcox	0.412780831187935			
141	2	0.5	Blue-negativeL_Putamen	wolf	wilcox	0.412780831187935			
141	1	1.0625	Blue-negativeL_Thalamus	wolf	wilcox	0.323335358781455			
141	2	0.333333333333333	Blue-negativeL_Thalamus	wolf	wilcox	0.323335358781455			
141	1	0.9375	Blue-negativeL_VentralD	wolf	wilcox	0.0669292770083106			
141	2	0	Blue-negativeL_VentralD	wolf	wilcox	0.0669292770083106			
141	1	0.1875	Blue-negativeR_Accumbe	wolf	wilcox	0.956043006882769			
142	2	0.166666666666667	Blue-negativeR_Accumbe	wolf	wilcox	0.956043006882769			
142	1	0.5625	Blue-negativeR_Caudate	wolf	wilcox	0.437805666932248			
142	2	0.833333333333333	Blue-negativeR_Caudate	wolf	wilcox	0.437805666932248			
142	1	1.3125	Blue-negativeR_Cerebell	wolf	wilcox	0.338830445401804			
142	2	0.5	Blue-negativeR_Cerebell	wolf	wilcox	0.338830445401804			
142	1	0.375	Blue-negativeR_Pallidum	wolf	wilcox	0.540666674412377			
142	2	0.5	Blue-negativeR_Pallidum	wolf	wilcox	0.540666674412377			
142	1	0.625	Blue-negativeR_Putamer	wolf	wilcox	0.42358870161628			
142	2	0.166666666666667	Blue-negativeR_Putamer	wolf	wilcox	0.42358870161628			
142	1	0.4375	Blue-negativeR_Thalamu	wolf	wilcox	0.827038122216554			
143	2	0.333333333333333	Blue-negativeR_Thalamu	wolf	wilcox	0.827038122216554			
143	1	0.625	Blue-negativeR_VentralD	wolf	wilcox	0.761506327066851			
143	2	0.333333333333333	Blue-negativeR_VentralD	wolf	wilcox	0.761506327066851			
143	1	5.75	Blue-negativeL_cortical..	wolf	wilcox	0.911347965290851			
143	2	5.33333333333333	Blue-negativeL_cortical..	wolf	wilcox	0.911347965290851			
143	1	7.125	Blue-negativeR_cortical..	wolf	wilcox	0.852994438248545			
143	2	6.5	Blue-negativeR_cortical..	wolf	wilcox	0.852994438248545			
143	1	6	Blue-negativeL_subcortic	wolf	wilcox	0.333329915232302			
143	2	3	Blue-negativeL_subcortic	wolf	wilcox	0.333329915232302			

143	1	4.125	Blue-negativeR_subcortic	wolf	wilcox	0.391971029871409			
144	2	2.83333333333333	Blue-negativeR_subcortic	wolf	wilcox	0.391971029871409			
